# Airborne observations over the North Atlantic Ocean reveal the importance of gas-phase urea in the atmosphere

**DOI:** 10.1073/pnas.2218127120

**Published:** 2023-06-14

**Authors:** Emily Matthews, Thomas J. Bannan, M. Anwar H. Khan, Dudley E. Shallcross, Harald Stark, Eleanor C. Browne, Alexander T. Archibald, Archit Mehra, Stéphane J.-B. Bauguitte, Chris Reed, Navaneeth M. Thamban, Huihui Wu, Patrick Barker, James Lee, Lucy J. Carpenter, Mingxi Yang, Thomas G. Bell, Grant Allen, John T. Jayne, Carl J. Percival, Gordon McFiggans, Martin Gallagher, Hugh Coe

**Affiliations:** ^a^Department of Earth and Environmental Science, Centre for Atmospheric Science, University of Manchester, Manchester M13 9PL, United Kingdom; ^b^Atmospheric Chemistry Research Group, School of Chemistry, Cantock’s Close, University of Bristol, Bristol BS8 1TS, United Kingdom; ^c^Center for Aerosol and Cloud Chemistry, Aerodyne Research, Inc., Billerica, MA 01821; ^d^Department of Chemistry, University of Colorado at Boulder, Boulder, CO 80309; ^e^Cooperative Institute for Research in Environmental Sciences, University of Colorado at Boulder, Boulder, CO 80309; ^f^Yusuf Hamied Department of Chemistry, University of Cambridge, Cambridge CB2 1EW, United Kingdom; ^g^National Centre for Atmospheric Science, University of Cambridge, Cambridge CB2 1EW, United Kingdom; ^h^Methods Analytics, London EC1N 8TS, United Kingdom; ^i^Facility for Airborne Atmospheric Measurements Airborne Laboratory, Cranfield University, Cranfield MK43 0AL, United Kingdom; ^j^Wolfson Atmospheric Chemistry Laboratories, Department of Chemistry, University of York, York YO10 5DD, United Kingdom; ^k^National Centre for Atmospheric Science, University of York, York YO10 5DD, United Kingdom; ^l^Plymouth Marine Laboratory, Plymouth PL1 3DH, United Kingdom; ^m^National Aeronautics and Space Administration Jet Propulsion Laboratory, California Institute of Technology, Pasadena, CA 91109; ^n^National Centre for Atmospheric Science, University of Manchester, Manchester M13 9PL, United Kingdom

**Keywords:** atmosphere, ocean, mass spectrometry, nitrogen cycling

## Abstract

Reduced nitrogen (N) is central to global biogeochemistry, yet there are large uncertainties surrounding its sources and rate of cycling. Here, we present observations of gas-phase urea (CO(NH_2_)_2_) in the atmosphere from airborne high-resolution mass spectrometer measurements over the North Atlantic Ocean. We show that urea is ubiquitous in the lower troposphere in the summer, autumn, and winter but was not detected in the spring. The observations suggest that the ocean is the primary emission source, but further studies are required to understand the responsible mechanisms. Urea is also observed aloft due to long-range transport of biomass-burning plumes. These observations alongside global model simulations point to urea being an important, and currently unaccounted for, component of reduced-N to the remote marine atmosphere. Airborne transfer of urea between nutrient-rich and -poor parts of the ocean can occur readily and could impact ecosystems and oceanic uptake of carbon dioxide, with potentially important climate implications.

## Significance

Reduced nitrogen (N) plays a fundamental role in ocean biogeochemistry, yet marine-reduced organic nitrogen (ON) species are poorly characterized as is their role in the global N cycle. Our observations suggest that biologically rich ocean environments are a significant source of urea to the atmosphere and that the atmosphere is likely to provide a fast transport route for the redistribution of reduced N across the seawater surface and as such have implications for marine productivity. Our findings show that the global marine burden of urea is significant which necessitates a revision of the atmospheric N cycle.

Concerns over the impacts of human perturbation to the atmospheric nitrogen (N) cycle have meant that inorganic N are reasonably well characterized, particularly oxidized N species (e.g., NO_x_) which has a substantial anthropogenic source. The importance of organic N (ON) has been realized over recent years, but the lack of knowledge as to its chemical composition has made it difficult to identify sources and sinks and to define atmospheric behavior (1). As a result, there are large uncertainties surrounding the role of ON in the global N cycle and to total atmospheric N deposition (2, 3).

In particular, ON in the remote marine environment has received far less attention compared with land-based sources of ON. The limited ON studies in this region have focused on water-soluble organic nitrogen in rainwater and aerosols (3–5) and have identified that urea (CO(NH_2_)_2_) makes a considerable contribution to the nutrient budgets of marine environments (6, 7). There remains however a significant gap in understanding how ON in remote environments contributes to the global N cycle.

The sources of urea are diverse with both anthropogenic and natural origins, but the magnitude and relative contribution of these sources are unclear. Natural sources primarily arise from marine biological activity (8), while anthropogenic sources include animals, fertilizers, industrial waste, and biomass burning (9–11). The presence of atmospheric urea in rainwater and aerosols has been associated with soil (7, 12, 13), anthropogenic activities (1, 6, 7, 14), marine environments (6, 12, 14), dust plumes (7, 14), and biomass-burning events (11).

In the remote marine atmosphere, urea has been predominately observed in fine-mode particulate aerosol, indicative of gas-to-particle conversion and that urea has the capability to be transported long distances (6, 7, 12–14). It has been suggested that a significant fraction of urea exists in the gas phase, as evidenced by a calculated dry deposition flux of particulate urea (0.59 mmol m^−2^) almost three times lower than the experimental value (1.78 mmol m^−2^), which may have been enhanced by gas-phase urea absorbing on deposited particles (7). Gas-phase urea in the atmosphere is possibly likely to be climatically important as theoretical studies have suggested its role in new particle formation (15–17), cloud condensation nuclei (18), ice-nucleating particle formation (19, 20), and the reduction of NO_2_ (18). Despite the evidence to suggest that atmospheric urea exists in the gas phase, there are as yet no ambient measurements to support this. This is in part due to the challenges in making such a measurement but also potentially due to an assumption that urea exists predominantly in the dissolved phase due to its high solubility.

However, equilibrium partitioning of urea between its aerosol and gas-phase states as a function of aerosol liquid water content (aLWC) suggests that approximately 80% of urea will exist in the gas phase, in a typical marine environment (*SI Appendix*, Fig. S15). It is known that urea can be produced in the near-sea-surface through metabolic activities of marine organisms, such as through phytoplankton, bacterial and viral metabolisms, zooplankton excretion, and cell death and decomposition (21–24). Outside of biological production, atmospheric deposition and terrestrial inputs can further supply urea to the sea surface (25, 26). Similar to biologically controlled compounds like dimethyl sulfide (DMS), its concentrations within the sea surface are highly variable and are a balance between its loss and production by marine organisms (27).

**Fig. 1.**
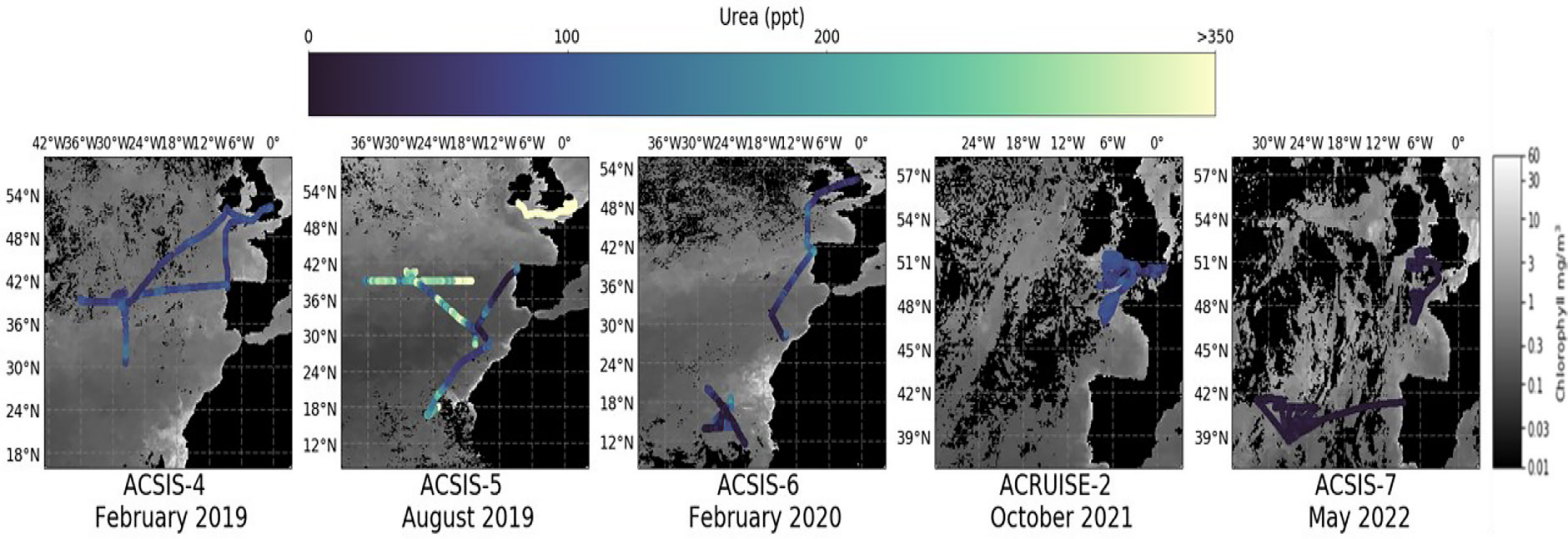
Overview of urea measurements during the ACSIS4-7 and ACRUISE-2 aircraft campaigns for all flight levels, which ranged from 0.03 to 8.2 km. The flight tracks are colored to the urea mixing ratios and overlaid onto gray-scale MODIS satellite monthly seawater chlorophyll data to indicate regions of biological activity.

**Fig. 2.**
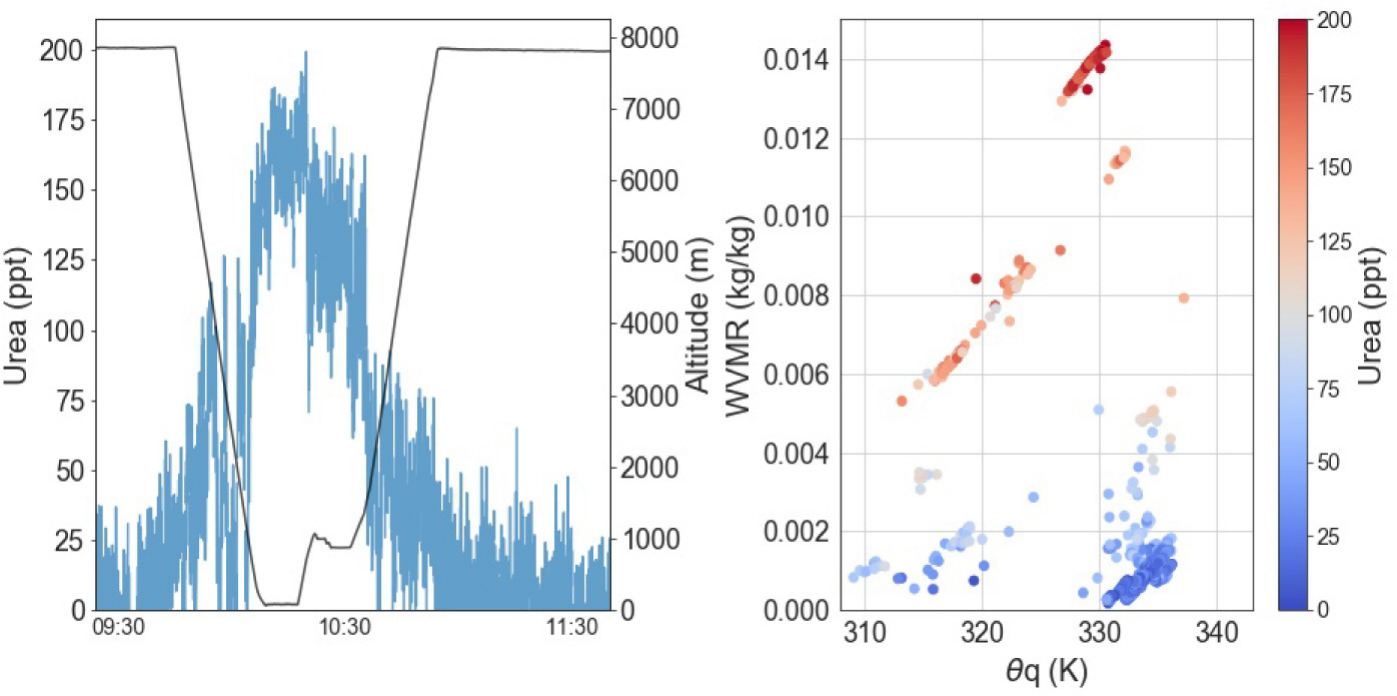
Example of measurements and thermodynamic analysis indicating that the ocean is a source of atmospheric gas-phase urea. Time series of urea mixing ratios during a flight across the North Atlantic showing enhancements of urea at the surface (*Left*). Thermodynamic analysis of water vapor mixing ratio (WVMR) against the wet equivalent potential temperature (Θq) for the measurements shown in the time series (*Right*). Analysis shows that the urea enhancements follow thermodynamically warm and moist air masses, indicating that the source is the sea surface.

**Fig. 3.**
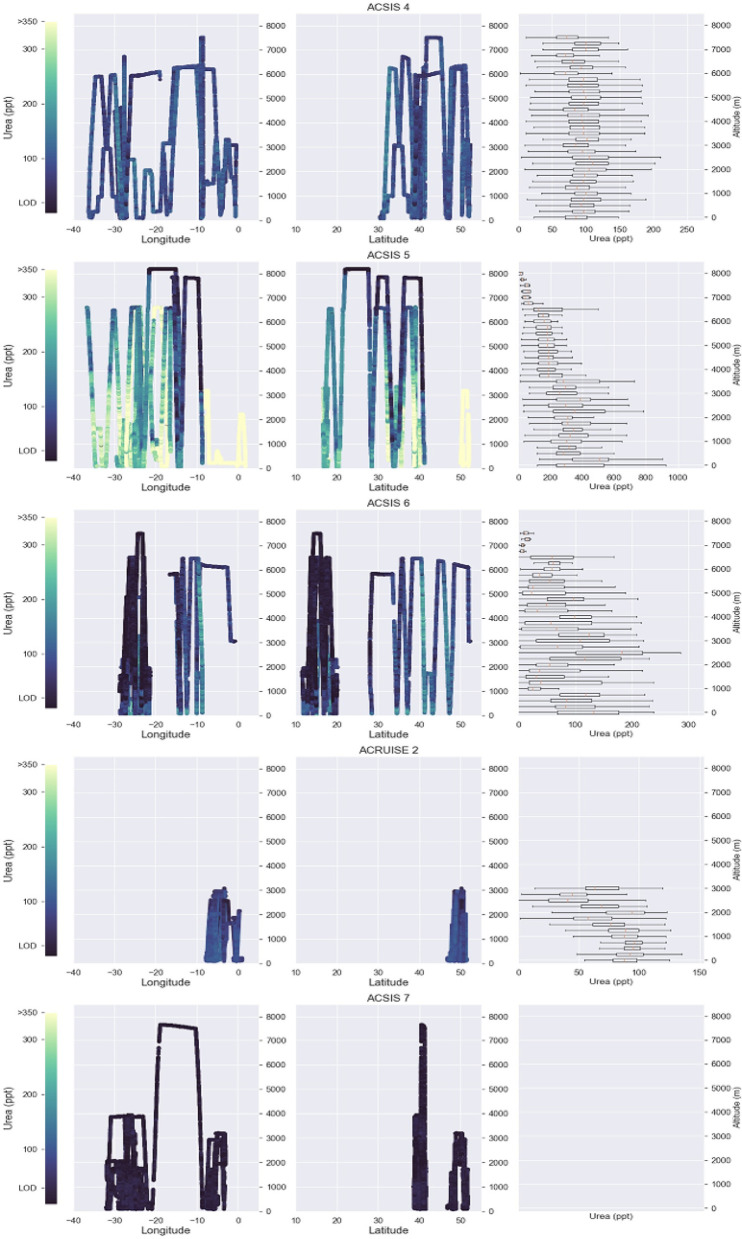
Profiles showing the spatial distribution of urea throughout the atmosphere during the ACSIS flights (ACSIS4-7; February 2019, August 2019, February 2020 and May 2022) and ACRUISE-2 flights (October 2021) The longitude, latitude, and adjacent average vertical profiles show the frequent occurrence of gas-phase urea throughout the atmosphere. The vertical profiles for the ACSIS-7 flights are not shown here as the measurements did not exceed the limit of detection. The vertical profiles show strong variability between each of the campaigns as a result of the different sources of urea and physical state of the atmosphere which impacts urea’s distribution.

**Fig. 4.**
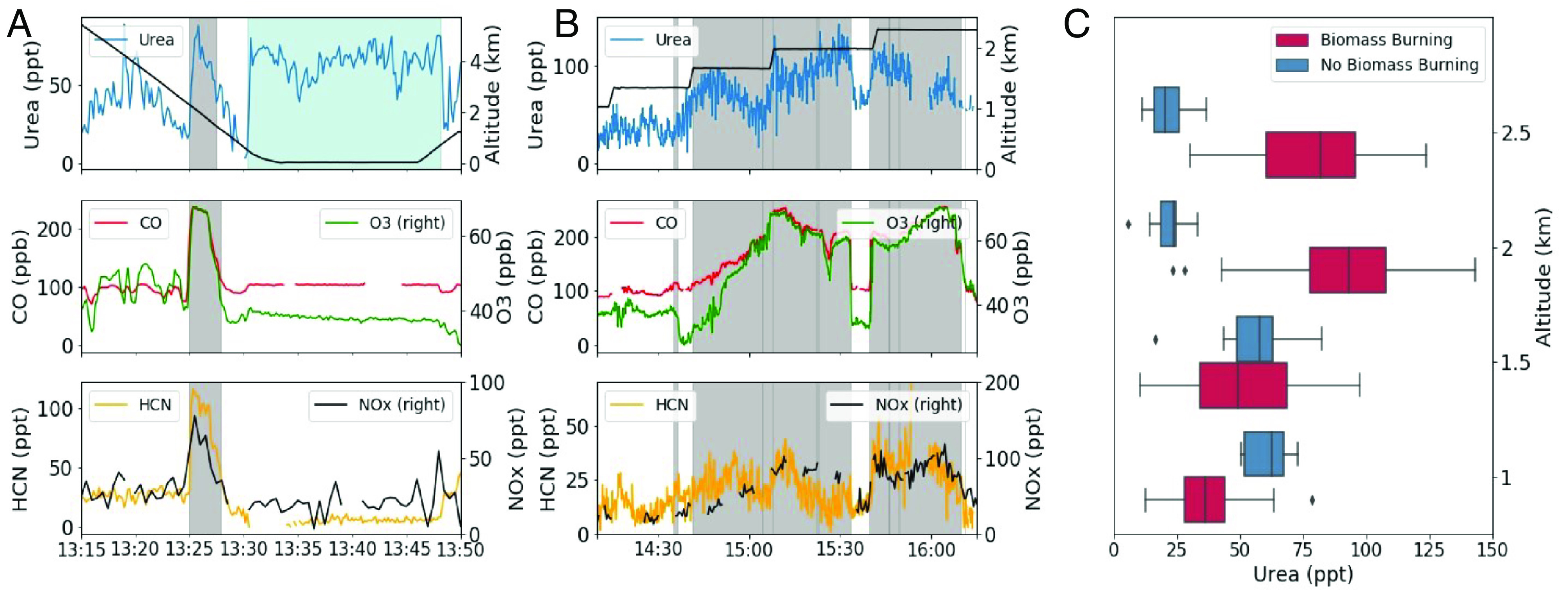
Observations of urea and other selected trace gases indicate biomass-burning inputs of urea to the marine environment. An example time series of urea concentrations is shown with the trace gases ozone (O_3_), carbon monoxide (CO), hydrogen cyanide (HCN), and nitrogen oxides (NO_x_) for a flight in the Cape Verde region in February 2020. (*A*) The blue shaded region indicates enhancement of urea at the sea surface due to biogenic sources. The gray shaded regions indicate periods when the aged biomass-burning plumes were intercepted and are above the BL. (*B*) shows enhancements of gas-phase urea in the free troposphere, which occurred exclusively in the presence of biomass-burning plumes. (*C*) The boxplot shows the urea concentrations within the FT for the time series show in this figure in red and are contrasted with measurements taken during a profile later in the flight, which encountered no biomass-burning plumes, in blue.

Here, we report gas-phase measurements of urea in the atmosphere using an airborne iodide ion high-resolution-time-of-flight-chemical ionization mass spectrometer (iodide CIMS). Our measurements show the frequent occurrence of gas-phase urea throughout the marine lower troposphere in significant quantities (Fig. 1). We show evidence that the marine environment is a significant source of gas-phase urea (Fig. 2) and that urea can be transported long distances (Figs. 3 and 4). Detailed modeling by a three dimensional global chemistry and transport model, STOCHEM-CRI (STOchastic CHEMistry-Common Representative Intermediates), demonstrates how this discovery impacts the global marine reduced N budget.

## Results

### A significant Ocean Source of Urea.

Flights were made over the North Atlantic Ocean as part of the ACSIS (North Atlantic Climate System Integrated Study) project (28) in Winter (ACSIS-4 & 6; February 2019 & 2020), Summer (ACSIS-5; August 2019), and Spring (ACSIS-7; May 2022) and as part of the ACRUISE (Atmospheric Composition and Radiative forcing changes due to UN International Ship Emissions regulations) project in Autumn (ACRUISE-2; October 2021). During these flights, urea was frequently observed throughout the marine lower troposphere and in air masses with low levels of pollutants and thus representative of the clean marine atmosphere (see *SI Appendix*, section 3 for more details) (Fig. 1). The urea mixing ratios were highest overall and most variable during the Summer flights, averaging 310 pptv and reaching a maximum of 1 ppbv in the remote central-North Atlantic region. The Winter and Autumn flights had similar mixing ratios, averaging 95 pptv and 80 pptv, respectively, although those originating from the tropical Atlantic region during ACSIS-6 reached over 200 pptv in some instances. The Spring flights had the lowest mixing ratios overall as all the measurements were below the limit of detection (<30 pptv).

Enhancements of gas-phase urea were frequently observed within the marine boundary layer (BL) across the North Atlantic basin and as such suggest a marine source. Air masses that originate from the surface ocean have warm and moist properties, which can be assessed using thermodynamic analysis of the wet equivalent potential temperature (*Θ*_q_) and measurements of water vapor mixing ratio (Fig. 2) (29). *Θ*_q_ is a property that is conserved during an air parcel’s transport through the atmosphere (i.e., as it experiences changes in pressure and is independent of the phase state of water). Urea was most abundant in warm and moist air masses which, combined with background levels of pollution tracers, indicate an ocean source. Our observations below 350 m do not show a dependency of the urea concentrations on physical variables such as temperature and wind speed, which suggests that atmospheric urea concentrations are governed more by local productivity than physical ocean–atmosphere exchange. However, comparison of the urea mixing ratios with satellite chlorophyll data suggests that chlorophyll is not a suitable indicator of urea emissions (Fig. 1).

### Effects of Local Atmospheric Conditions on the Spatial Distribution of Urea.

The higher urea mixing ratios observed during the summer extended beyond the BL to the free troposphere (FT), where mixing ratios were often double that of the Winter flights, typically exceeding 200 pptv (Fig. 3). At a regional scale, the vertical distribution appears to be driven by the local physical state of the atmosphere and is consistent with a trace gas originating from the sea surface. When anticyclonic conditions dominate over the subtropical Atlantic Ocean, a strong inversion at the BL top persists. Under these conditions, we observed that elevated urea concentrations were constrained to the BL below the inversion top (*SI Appendix*, Fig. S13*A*), with mixing ratios ranging from 100 to 350 pptv.

Similar profiles were observed over the tropical eastern North Atlantic Ocean during the winter months where low altitude biomass burning and dust outflow from continental Africa, found above the BL, dominate the region (30, 31). In this region, we observed the surface layers to be minimally influenced by biomass burning and anthropogenic inputs, as indicated by low NO_x_, CO, O_3_, and hydrogen cyanide (HCN) concentrations, and as such representative of a clean marine BL. The BL top layer was again characterized by a sharp capping inversion resulting in a confined BL (*SI Appendix*, Fig. S13*B*). Thus, the probable source of urea within the BL, where mixing ratios ranged from 50 to 250 pptv, was the sea surface. However, unlike during other periods of the year, additional enhancements were observed in the FT in the wintertime and are discussed subsequently.

To a first approximation (*SI Appendix*, section 3), the time taken for one turnover of the BL is less than 30 min. Therefore, the presence of urea throughout the BL under capped conditions points to its lifetime being substantially longer than 30 min in the clean marine environment. In other instances, such as in the presence of a frontal passage which enables air to be rapidly lifted and mixed through the atmosphere, deeper mixing was observed. This feature was particularly dominant during ACSIS-4 (February 2019) where a high-speed jet stream was prevalent across the North Atlantic. Under these conditions, we observed urea to be well mixed throughout the atmospheric column up to the maximum altitude sampled of approximately 7.5 km (Fig. 3).

### Enhancements in the FT due to Biomass Burning.

While the clean marine environment appears to act as an important source of the elevated urea concentrations observed, there are some instances of occasional enhancements in the FT that point to an additional source. During ACSIS-6 (February 2020), we frequently observed episodic enhancements of urea in both the BL and FT over the tropical eastern North Atlantic. Whereas the enhancements in the BL were associated with clean air and marine characteristics, above the BL, the atmosphere was disturbed by discrete biomass-burning layers from continental Africa, indicated by enhancements in NO_x_, CO, O_3_, and HCN concentrations and back trajectory analysis (*SI Appendix*, section 3). Enhancements of gas-phase urea in the FT were observed exclusively in the presence of these plumes demonstrating biomass burning as an additional source. The urea concentrations in the biomass-burning plumes were on average 63% higher than in nonpolluted air masses at similar altitudes. The biomass-burning plume concentrations were, however, lower than the urea concentrations observed in the BL (*SI Appendix*, Fig. S14).

Fig. 4 demonstrates the unequivocal observation of biomass burning–derived urea during ACSIS-6. The gray shaded regions indicate periods when biomass-burning plumes were intercepted and were identified using a statistical threshold approach (*SI Appendix*, section 3). Fig. 4*A* shows enhancements in urea observed during a profile to the surface and as the aircraft intercepted a biomass-burning plume just above the BL. A subsequent enhancement was then observed at the surface, originating from the sea surface (indicated by the blue shaded region in Fig. 4*A* demonstrating the clean state of the marine BL). Panel (b) shows observations from straight level runs within the FT during periods when urea was enhanced. The urea concentrations within the FT from the time series shown in panel (a and b) are shown in Fig. 4*C* in red and highlight the urea enhancements observed aloft in biomass-burning plumes. This is contrasted with urea concentrations from a profile later on in the flight in blue, which encountered no biomass-burning influence and showed no enhancement of urea in the FT. A strong correlation with HCN, a unique biomass-burning marker, was not observed with the urea enhancements we link to biomass burning, but this may be due to the age of the plumes (32) and the likely different sinks of urea and HCN. The age of these air masses has been previously estimated in another study using the ratio of benzene:CO to be 147 to 201 h (32).

Furthermore, to determine the magnitude of biomass-burning sources of urea, we analyzed measurements made during a separate project, MOYA-II (Methane Observations and Yearly Assessments), where fresh fire plumes over East Africa were directly sampled. During fire plumes sampled over Uganda, enhancements in urea were observed. In this region, the dominant fuel type is assumed to be from savannah and grassland burning. Compared with other N species emitted from the burning of this fuel source, the calculated emission ratio enhancement factors for urea are considerably smaller (*SI Appendix*, section 3). However, biomass-burning may still be an important source of urea, particularly since we show that urea can be found in aged biomass-burning plumes far from the source. On the contrary, no enhancements in urea were observed during fire plumes sampled over the Kafue wetland, Zambia, which are suspected to have a similar fuel source to fires sampled over Uganda. It is possible that urea is not emitted from fire emissions themselves, but rather urea is associated with the emissions of soil that have been elevated in the strong updraughts generated in the nearfield of the fires. Intensive fires are known to remobilize soil minerals and organics from the ground (33, 34).

### Exploring the Processes Controlling the Air–Sea Gas Exchange of Urea.

The lack of understanding of the role of urea within our Earth system is largely due to the expectation that its gas-phase abundance is insignificantly small, not only within the marine atmosphere but globally (35). Our observations show that this assumption may not be correct. We examine the potential impacts of a significant presence of gaseous urea in the atmosphere using a global chemistry and transport model, STOCHEM-CRI. Though at the present time, many of the details of the behavior of urea in the atmosphere are poorly understood, our model approach assumes that urea has a marine emission similar to that of DMS, behaves in a similar way to ammonia (NH_3_) in the atmosphere and is removed at a similar rate, and has emissions tuned to match observations. This serves to provide an initial indication of the global marine burden of urea and its contribution to total reduced N and points to the need for a more detailed investigation of many components of urea in the marine atmosphere.

Our observations suggest that the ocean can act as a significant emission source; thus, the surface distribution for urea emissions from the ocean implemented in the model is based on that of DMS (36). We used 56.2 Tg N/yr gas-phase urea emissions from ocean in the model, which is twofold higher than that used for DMS, and was chosen for its overall reasonable agreement with our observations of atmospheric BL gas-phase urea concentration (*SI Appendix*, section 2). The loss fluxes of urea are also unknown; thus, the model parameters related to physical loss processes of urea implemented in the model are based on those of NH_3_. In order to sustain a direct flux of 56.2 Tg N/yr of gas-phase urea, the dissolved concentrations of urea in the near-sea-surface would need to be approximately 170 mmol L^−1^ based on the “two-film” model for air–sea gas exchange (37). This is many orders of magnitude higher than the current reported measurements of 300 nmol L^−1^ for open ocean (*SI Appendix*, section 3) (27).

We find that our gas-phase measurements are consistent with equilibrium partitioning based on previous measurements of particulate urea in the marine atmosphere (38) and typical liquid water content of marine aerosols (aLWC) (*SI Appendix*, section 3). A lower aLWC results in a greater fraction of urea in the gas phase and as such suggests that under dry conditions and low relative humidity, most of the urea is in the gas phase. Conversely, at humidities above 95% and in cloud, atmospheric urea exists almost exclusively in the aqueous phase. However, despite being consistent with the limited available measurements of aerosol urea, the concentration of aerosol urea is inconsistent with expectations based on sea surface concentrations (*SI Appendix*, section 3). Emissions of nonvolatile compounds are often associated with bubble bursting, a process whereby organic material within the sea surface is ejected as particulate matter along with sea spray into the atmosphere. Strong surface enhancements of urea, approximately 3 to 30 mmol L^−1^, would be required to sustain the atmospheric observations from bubble-mediated processes (*SI Appendix*, section 3). Again, this is several orders of magnitude higher than the current reported concentrations of urea in open ocean.

The inconsistencies between the atmospheric and ocean observations highlight the urgent need for more detailed measurements of the processes and interactions occurring within the sea surface. In particular, despite being the ultimate interface that governs exchange between the ocean and air, the sea-surface microlayer (SML) is poorly understood. The water-side layer in particular shows distinct physical, chemical, and biological properties (39). Therefore, the urea concentrations within this layer may be higher than current reported measurements for the near-surface water. It is also possible that there are interactions occurring between urea and other compounds within the SML that increase urea’s volatility.

### Global Marine Burden of Urea.

Our model results have been used only to indicate the likely size of the global burden of gas-phase urea from marine emissions based on our observations. The annual tropospheric global marine burden and lifetime of gas-phase urea are found to be 0.16 Tg N and 1.0 d, respectively. Using the same global model, we have also determined the tropospheric global marine burden of NH_3_ (0.021 Tg N). Thus, if urea does behave in a similar manner to NH_3_, then it is the dominant reduced N species in the marine environment and can increase the marine reduced N burden by ~350% (*SI Appendix*, section 2). Compared with the total global burden of NH_3_ (0.23 Tg N) (40)^,^ the results are still significant. These preliminary results indicate the necessity for future work as the implications could be very significant if the initial model simulations of the oceanic reduced N burden reflect those in the real world.

### Current Uncertainties and Future Outlook.

Based on our observations, the atmosphere is very likely to provide an important but previously overlooked pathway for distributing reduced ON throughout the Earth system. However, there is a need for more research to understand the mechanisms responsible for the significant presence of gas-phase urea in the atmosphere. Furthermore, the global source strength of urea, the kinetics for the reaction of urea with the oxidants, and the depositional parameters (e.g., deposition velocity, scavenging coefficients) need to be reevaluated to understand and constrain the role of urea in the reduced N cycle.

Nonetheless, the influence of reduced N to the remote marine environment could therefore be greater than currently thought. Although anthropogenic NH_3_ emissions have led to a substantial increase in NH_x_ concentrations over recent decades, the relatively short atmospheric lifetime of NH_x_ often prevents significant concentrations reaching remote marine environments (41). The simulated loss of urea via dry and wet deposition is such that urea can be transported significant distances and supports the observations that point to urea being a major source of reduced N to remote regions. Furthermore, our calculations that show the dependency of urea partitioning with aLWC suggest that, in the absence of precipitating cloud and hence no wet removal, loss will be via dry deposition to the surface ocean in regions where there is a urea deficit in the surface water compared to the gas phase. Emissions of urea from biologically rich ocean environments therefore provide a pathway for broad fertilization of the surface ocean at rates and at scales far greater than could be possible from ocean transport, particularly since we show that urea may be lifted into the FT via frontal transport and so transported significant distances. We have observed urea in the marine environment in polluted air masses aged 6 to 8 d, also providing evidence for long-range transport of polluted air masses being an additional important pathway due to urea’s relatively long atmospheric lifetime.

In our simulations, we have considered an exclusive ocean source of urea. Considering also anthropogenic sources, the contribution of reduced N from urea will likely increase. However, more detailed observations are required to refine the model. In particular, detailed observations of likely major anthropogenic sources are required. Worldwide use of urea has increased dramatically over recent decades and now constitutes >50% of global nitrogenous fertilizer use and thus fertilizers, along with livestock waste, could be a large source of urea to the atmosphere. Coincidently, observations of urea in rainwater and aerosols are currently highest for samples collected over the East China Sea, a region which dominates the global consumption of urea (42) and as such may elucidate to the global distribution of urea (14, 43).

Our observations provide important evidence on the presence of gas-phase urea in the Earth’s atmosphere. These data suggest that urea is an important source of the reduced N budget of the lower atmosphere which may lead to a significant revision of the global N cycle since there are important implications for our current understanding of how N deposition over oceans impacts ecosystems and the oceanic uptake of carbon dioxide.

## Materials and Methods

Airborne observations of gas-phase urea were collected onboard the UK Facility for Airborne Atmospheric Measurements (FAAM; https://www.faam.ac.uk/) BAe-146 atmospheric research aircraft. Measurements were made with an iodide CIMS during several campaigns over the North Atlantic Ocean and East Africa. Urea is detected as a stable adduct with iodide (I-CH_4_N_2_O) at m/z 186.937383. This peak is in close proximity to the organic acid acetic acid but is resolvable with accurate mass calibrations and a high-resolution instrument. This is however specific to the instrument used in this study and as such care should be taken when using other systems. The measurements were calibrated to give humidity-corrected and quantitative concentrations (pptv). Further details on the instrument operation, data analysis, and calibration methods for the iodide CIMS measurements of urea and supporting trace gas measurements are provided in *SI Appendix*, section 1.

Global model simulations of urea were generated using the STOCHEM-CRI model. As there are no emission inventories for urea, the oceanic spatial and temporal variation was simulated using that of DMS emissions. The depositional parameters (deposition velocities over land and ocean, scavenging coefficients) of dry deposition and wet deposition of urea used in the model are assumed to be the same as ammonia. Details on the STOCHEM-CRI model simulations are provided in *SI Appendix*, section 2.

## Supplementary Material

Appendix 01 (PDF)

## Data Availability

Aircraft measurements will be available on the CEDA Archive in the individual flight folders from the ACSIS (https://catalogue.ceda.ac.uk/uuid/7e92f3a40afc494f9aaf92525ebb4779) (44), ACRUISE (https://catalogue.ceda.ac.uk/uuid/d6eb4e907c124482881d7d03c​06903e4) (45), ARNA (https://catalogue.ceda.ac.uk/uuid/1a7f76b65e774cf6abb09cdee642dd73) (46), and MOYA projects (https://catalogue.ceda.ac.uk/uuid/dd2b03d085c5494a8cbfc6b4b99ca702) (47).
