# Supplementary material for: Airborne observations over the North Atlantic Ocean reveal the importance of gas-phase urea in the atmosphere
Source: Proc Natl Acad Sci U S A. 2023 Jun 14;120(25):e2218127120. doi: 10.1073/pnas.2218127120 (PMC10288635; doi:10.1073/pnas.2218127120)
Supplement: Supplementary file 1 — Appendix 01 (PDF) [file pnas.2218127120.sapp.pdf]

Supplementary Information

Section 1: Instrument Operation and Calibration

Campaign Overview

Airborne measurements of urea ($CO(NH_2)_2$) were recorded over the North Atlantic Ocean as part of the ACSIS (North Atlantic Climate System Integrated Study) aircraft campaign in February 2019, August 2019, February 2020 and May 2022 and as part of the ACRUISE (Atmospheric Composition and Radiative forcing changes due to UN International Ship Emissions regulations) aircraft campaign in October 2021 and May 2022. Additional measurements were collected over Uganda as part of the MOYA (Methane Observations and Yearly Assessments) project during an aircraft campaign in January 2019. The UK Facility for Airborne Atmospheric Measurements (FAAM; https://www.faam.ac.uk/) BAe-146 atmospheric research aircraft was used for all these projects.

ACSIS

The ACSIS project (http://acsis.ac.uk/) focuses on understanding recent changes in the ocean, the atmosphere, the cryosphere and the interactions between each of these components that make up the North Atlantic climate system. The data presented here was collected during four aircraft campaigns designed to observe recent changes in atmospheric chemistry, primarily with processes occurring in the gas-phase. The measurements given here were taken during campaigns covering the boreal spring, summer and winter; ACSIS-4 (19-22nd February 2019, C139-C145), ACSIS-5 (13-22nd August 2019, C199-C211), ACSIS-6 (3-14th February 2020, C215-C224) and ACSIS-7 (3rd-8th May, C288-C293). The flights during the ACSIS campaign concentrated on the eastern side of the North Atlantic basin, flying between 52°N and 12°N, covering regions over and between the United Kingdom (UK) and Republic of Ireland (R. Ireland), Azores, Canary Islands and Republic of Cape Verde. The ACSIS-5 and ACSIS-6 campaigns coincided with the NERC ARNA project (Atmospheric Reactive Nitrogen over the remote Atlantic) which focuses on sources of NO_x in the remote marine troposphere. Flights C206-C209 (ACSIS-5) and C218-C224 (ACSIS-6) were funded as part of the ARNA project but are referred to as ACSIS in this study for simplicity.

ACRUISE

The ACRUISE project aims to quantify the effect of changes in atmospheric chemistry and radiative forcing as a result of International Maritime Organisation (IMO) regulations that requires ships in international waters to reduce their SO_2 emissions from a maximum of 3.5% to 0.5% from January 2020. The data presented here was collected during the ACRUISE-2 (28th September-7th October, C254-C261) and ACRUISE-3 (30th April-2nd May, C285-C287) aircraft campaign. The flights during the ACRUISE campaign concentrated off the coast of the UK and France, primarily focussing on busy shipping lanes and sulfur emission control areas (SECA). The ACRUISE-3

and ACSIS-7 flights were a joint campaign and all flights as part of these two projects are referred to as ACSIS-7 in this study for simplicity.

MOYA-II

The MOYA-II campaign (flight numbers C127-C137) took place in early 2019 in Uganda (24-29th January 2019) and Zambia (1st-3rd February 2019). The objective of the MOYA project is to move towards closing the global methane budget through the undertaking of new observations and further analysis of existing data. The flights used in this study include only those that encountered biomass burning events; C132, C133 C134 and C137. Flight C132 was intended to survey the biogenic methane emissions from Lake Kyoga and the surrounding wetlands but also included sampling of some biomass burning events that were found to be occurring at the edges of Lake Kyoga. Flights C133 and C134 were designated to target biomass burning in the north-west of Uganda. All the flights over Uganda took off and landed from Kampala airport, located north-west of Lake Victoria. Flight C137 was designed to measure CH_4 emission fluxes from different parts of the Kafue wetland but also sampled some biomass burning events.

Application of an iodide HR-ToF-CIMS for gas-phase urea measurements

In-flight Sampling

The University of Manchester High Resolution-Time of Flight-Chemical Ionisation Mass Spectrometer (ToF-CIMS) that has been described in detail by (1, 2) for ground based deployment has been modified and certified for use on the FAAM Research Aircraft and was used for real time detection of urea in this study. The original instrument was manufactured by Aerodyne Research Inc. and employs the ARI/Tofwerk High Resolution Time of Flight Mass Spectrometer. Briefly, iodide ions cluster with sample gasses in the ion-molecule reaction region (IMR) region creating a stable adduct. The flow is then sampled through a critical orifice into the first of the four differentially pumped chambers in the TOF-CIMS, the short segmented quadrupole (SSQ). Quadrupole ion guides transmit the ions through these stages. The ions are then subsequently pulsed into the drift region of the ToF-CIMS where the arrival time is detected with a pair of microchannel plate detectors with an average mass resolution of 4000 (m/Δm).

The inlet design was based on the configuration characterised by (3), an atmospheric pressure, rearward facing, short residence time inlet, consisting of 3/8" diameter polytetrafluoroethylene (PTFE) tubing with a total length to the instrument of 48 cm. A constant flow of 12 SLM is mass flow controlled to the ion-molecule reaction region (IMR) using a rotary vane pump (Picolino VTE-3). 1 SLM is then subsampled into the IMR for measurement.

An Iris system as described by (4) was employed to pressurize and mass flow control the sample flow into the instrument, avoiding sensitivity changes that would be associated with variations in pressures inflight that is not controlled sufficiently by the constant flow inlet. This works upon the principle of the manipulation of the size of the critical orifice in response to changes in the IMR pressure. As with the (4) design, this works by having a stainless steel plate with a critical orifice and a movable PTFE plate on top of this, also with a critical orifice.

These orifices either align fully and allow maximum flow into the instrument or misalign to reduce flow. This movement is controlled by the 24VDC output of the IMR Pirani pressure gauge in relation to the set point and was designed collaboratively with Aerodyne Research Inc. The IMR set point was 72±3 mbar for the aircraft campaigns which is set through a combination of pumping capacity on the region (Agilent IDP3), mass flow controlled reagent ion flow and sample flow. The reagent ion flow is 1 SLM of ultra-high purity (UHP) nitrogen mixed with 2 SCCM of a pressurized known concentration gas mix of CH_3I in nitrogen, passed through the radioactive source, ^{210}Po. The total flow through the IMR is measured (MKS MFM) at the exhaust of the Agilent IDP3 pump so that not only the IMR pressure is monitored but the sample flow also. All mass flow controllers and mass flow meters are measured and controlled using the standard Aerodyne Inc EyeOn control unit and software.

A pressure controller is also employed on the short segmented quadrupole (SSQ) region to make subtle adjustments in this region independently of any small IMR changes that may occur inflight. This works upon the principle controlling an electrically actuated solenoid valve in a feedback loop with the SSQ pressure gauge to actively control a leak of air into the SSQ pumping line. The SSQ is pumped using Ebara PDV 250 pump and held at 1.8±0.01 mbar.

Instrument backgrounds are programmatically run for 6 seconds every minute for the entire flight, by overflowing the inlet with ultra high purity (UHP) nitrogen at the point of entry into the IMR. Here a 1/16th inch PTFE line enters through the movable PTFE top plate, ensuring that the flow exceeds that of the sample flow. Inlet backgrounds are also run multiple times during campaigns manually by overflowing as close to the end of the inlet as possible with UHP nitrogen. Data is taken at 4Hz during a flight, which is routinely averaged to 1 Hz for analysis. Of the 6 points in each background, the first 2 and last point are unused and the mean of the background is calculated using custom python scripting. Backgrounds are humidity corrected and using linear interpolation, a time series of the instrument background is determined and then subtracted to give the final time series of urea. The time series indicates the fast measurement response of the CIMS to this species (*Figure S1*). Inlet characteristics were studied in detail to exclude any effects on the urea signal and the instruments (*Figure S2-3*).

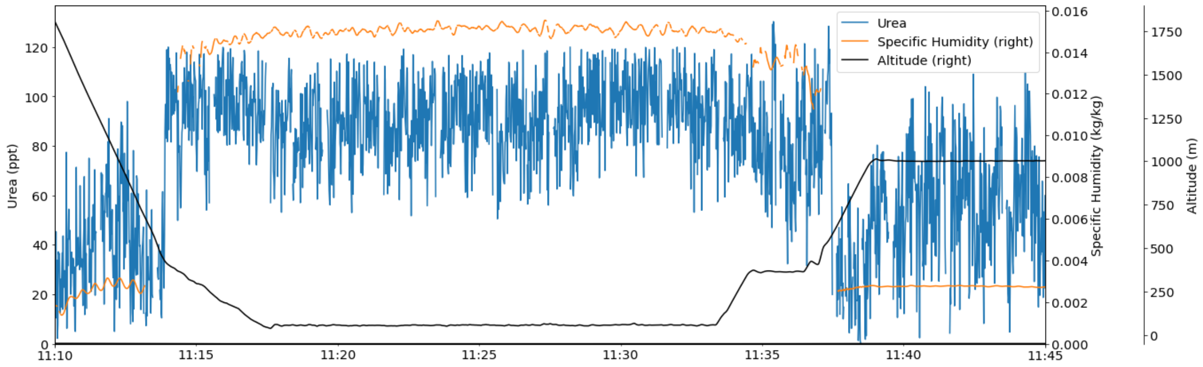

Figure S1: Example 1 Hz time series from a flight showing the fast response of the urea signal to changes in atmospheric humidity. The example shown is for a flight during a surface layer run in a confined MBL and where the enhancement is due to ocean emissions. The immediate response of the urea signal to sampling in and out of the BL, indicates the fast measurement response of the CIMS to this species.

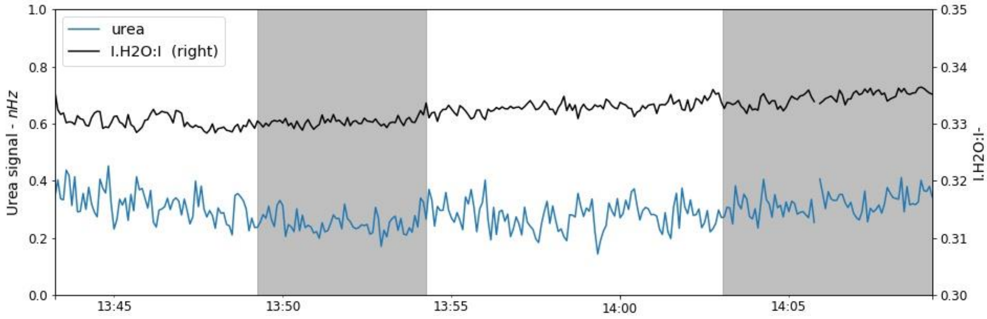

Figure S2: Ambient air sampled with cycles of the aircraft inlet being attached on/off to the iodide-CIMS. The CIMS was configured to replicate the conditions used on the aircraft. Grey regions indicate periods with the inlet being attached to the instrument. Cycles show no direct inlet effect when not varying relative humidity.

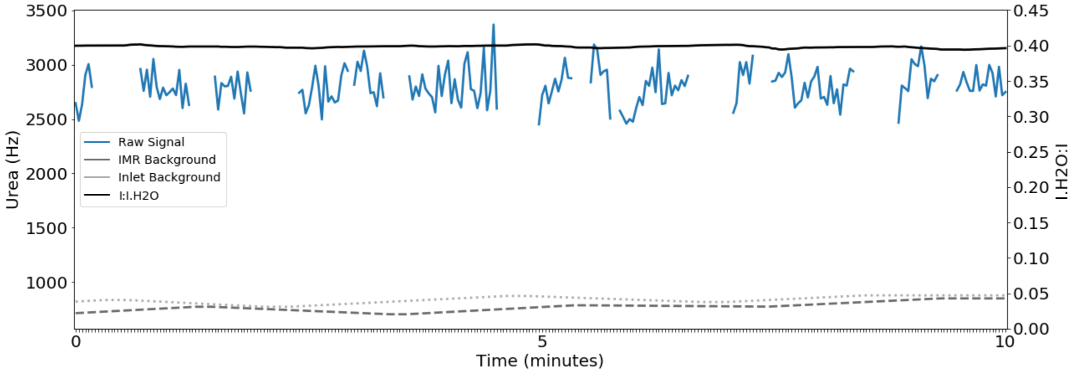

Figure S3: A comparison of the aircraft inlet and instrument background under ambient conditions. Prior to the measurements the inlet system underwent a period of “urea soaking” where urea produced through the bubbler was passed into the inlet system.The CIMS was configured to replicate the conditions used on the aircraft. The results indicate that the inlet background levels are similiar to the IMR background levels, which are used to background correct the flight measurements.

Peak Identification and Fitting

The CIMS instrument analysis software (ARI Tofware version 3.1.0, (5))was utilized to attain high resolution, 1Hz, time series of the compounds presented here. Mass-to-charge calibration was performed for 5 known masses; I-, I-.H_2O, I-.HCOOH, I_2-, I_3-, covering a mass range of 127 to 381 m/z. The mass-to-charge calibration was fitted using the square-root equation and was accurate to within 3 ppm.

Urea is detected as a cluster with iodide (I-CH_4N_2O) at an *m/z* of 186.937383. This peak is within close proximity (60 ppm, 0.5 HWHM) to the organic acid acetic acid at 186.926150 *m/z* (*Figure* S4), and as result introduces additional uncertainties for the peak area. Diagnostics associated with multi-peak fitting, based on (6) were performed using the analysis software (*Figure S5*). The uncertainty from the accuracy of the multi-peak fitting was determined to be 0.5-5 % depending on the relative intensities of the two peaks. The low uncertainties associated with the multi-peak fitting demonstrate the sufficient resolving power of the CIMS used in this study to separate the two peaks. This calculated uncertainty is however unique to this instrument and rely on accurate mass calibrations and so may differ for other I-CIMS instruments. The uncertainty from the mass-to-charge calibrations, calculated at an average error of 3 ppm, gave an additional intensity inaccuracy of 6-10%. Combined this gives a largest uncertainty of 6-11%, with most of this uncertainty arising from the mass calibrations.

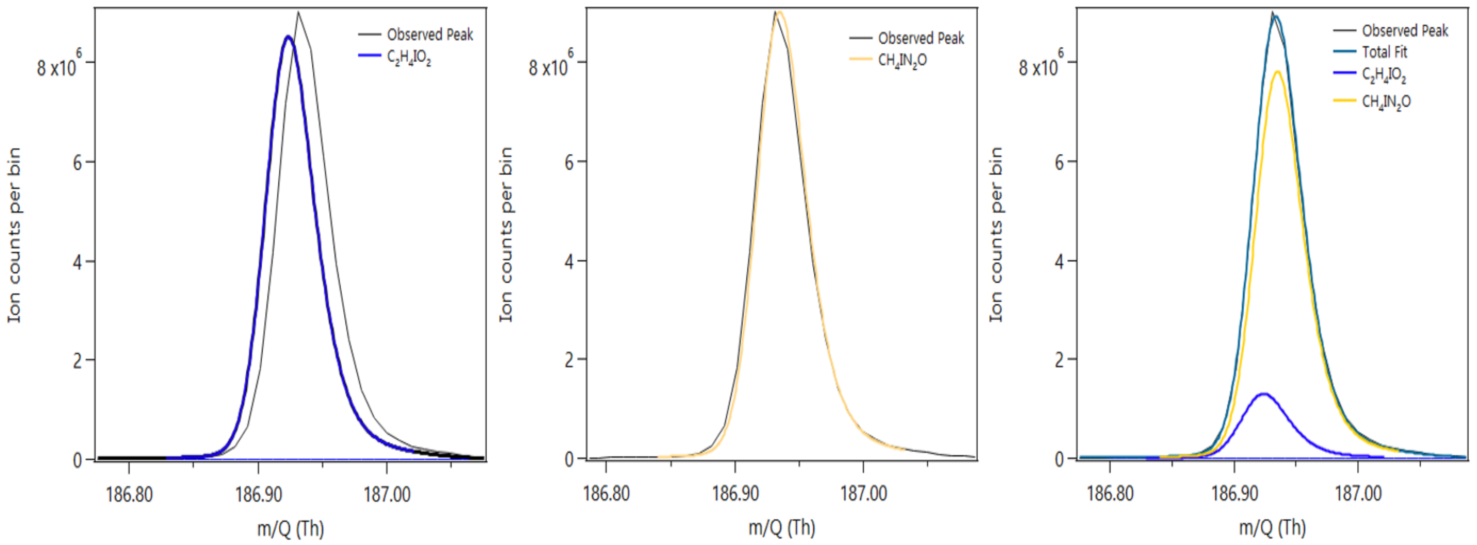

Figure S4: Peak fitting of acetic acid (left), urea (middle) and both species (right) at m/z 187. The mass defect data shows that in our spectrum urea dominates the peak at m/z 187. A sample of data from all the flights detailed in this study was used to generate the example mass spectrum.

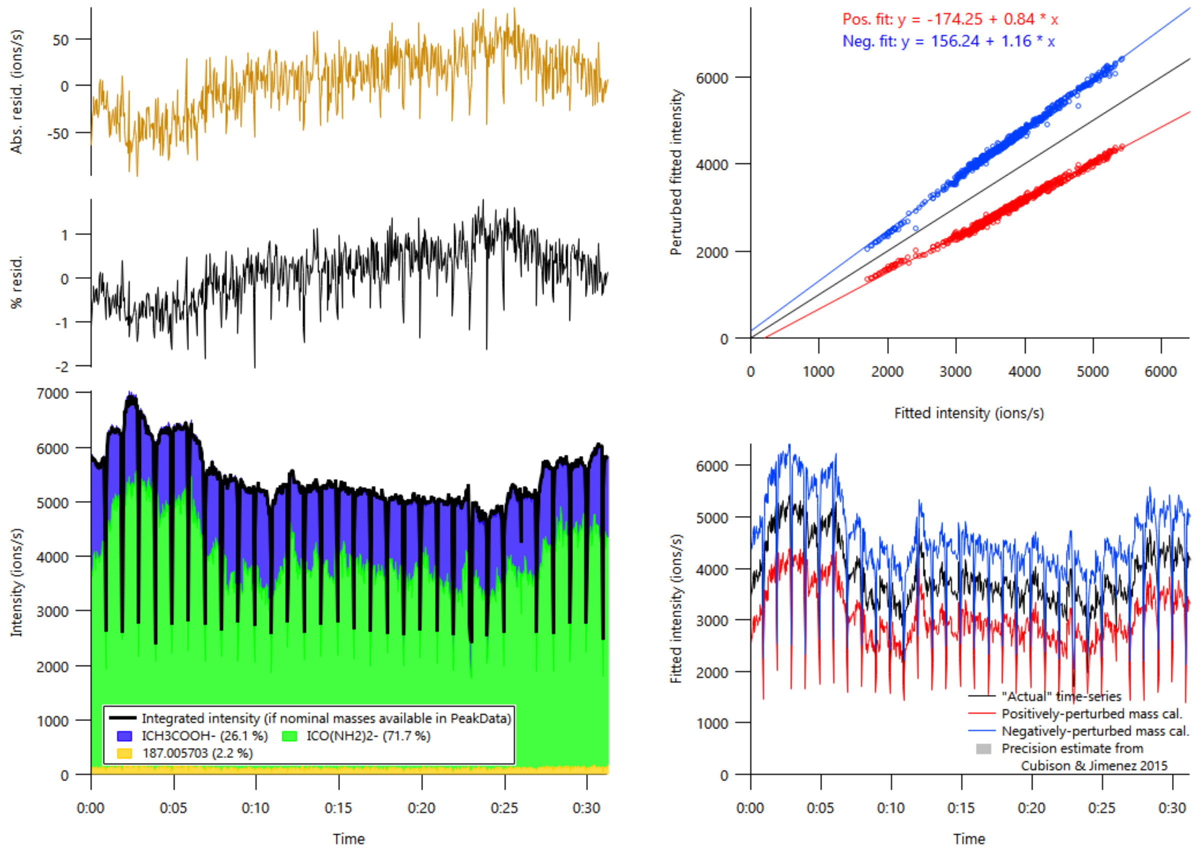

Figure S5: Diagnostics associated with multi-peak fitting, based on Cubison and Jimenez (2015) performed using the analysis software. Top left panel shows that the residuals are around zero, indicating no missing additional peaks. Top right panel is the scatter plot of the uncertainties due to mass calibration fluctuations of up to 5 ppm. Bottom left panel shows the contribution of all ions contributing to the signal at unit mass 187 to the total signal. Bottom right panel is the time series of the original and m/z perturbed (by 5 ppm) urea signal. The precision estimate from Cubison & Jimenez (2015) was determined to be 0.5-5 %.

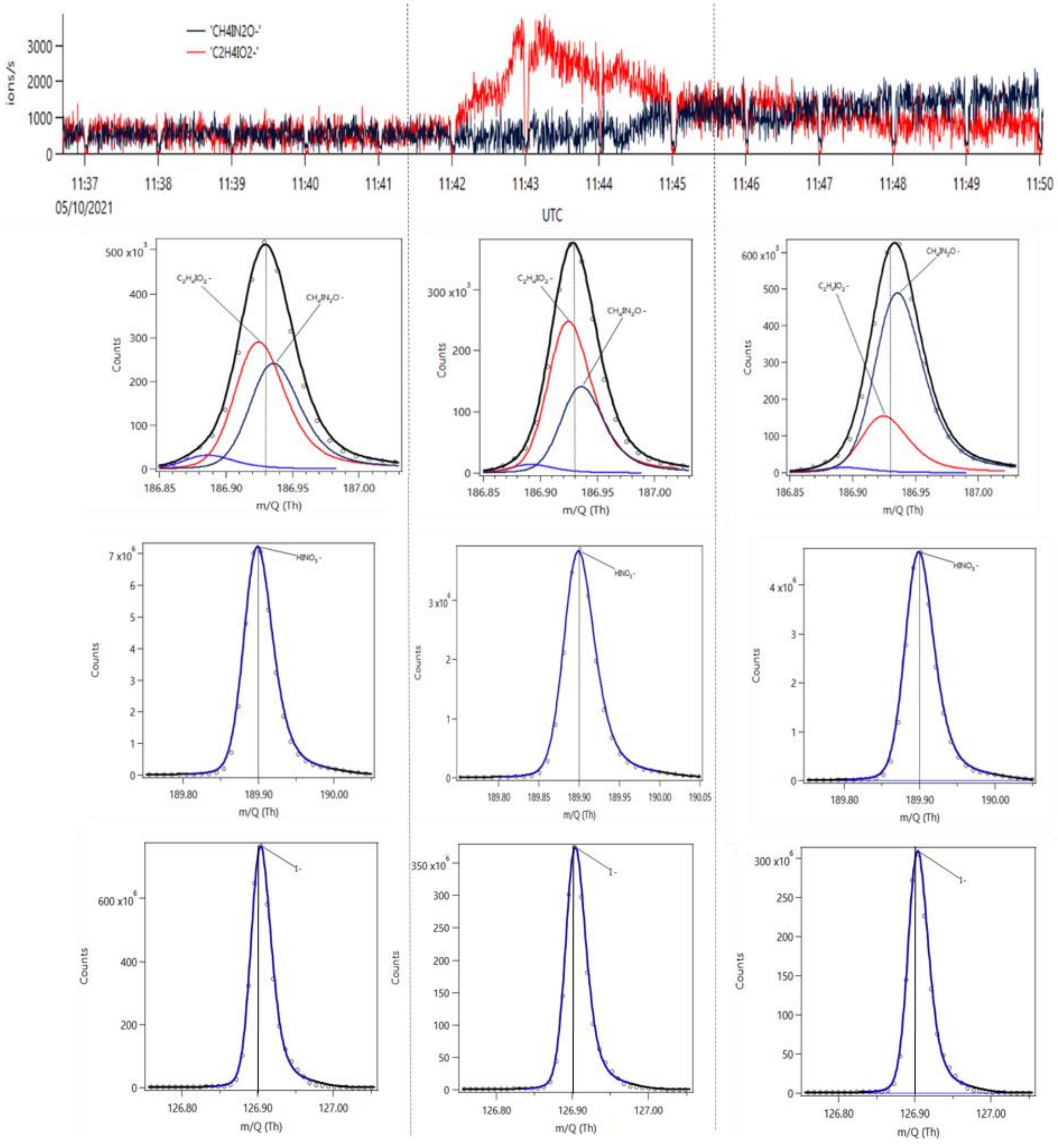

Figure S6: Example time series during ACRUISE-2 demonstrating the capability of the CIMS used in this study to resolve the acetic acid and urea peak at m/z 187. The black dashed line is plotted at 186.93 m/z to demonstrate the peak shift as a result of the change in intensity of the urea and acetic acid signals. The nearby HNO_3 peak at m/z 190 and I^- peak at m/z 127 are also shown to demonstrate the stability of the mass calibrations at this time.

Calibration and Sensitivity Determination

Two methods were combined to determine the instrument sensitivity and relative humidity dependence of gas-phase urea measurements. The Filter Inlet for Gases and Aerosols (FIGAERO) coupled to the CIMS was utilised to determine quantitative sensitivity values by volatilising a known concentration of urea from a filter (7, 8). This is the first study to demonstrate the utility of a FIGAERO-CIMS to determine humidity-dependent sensitivities. The humidity dependence was then verified using a series of *bubbler experiments* where a constant flow of urea was produced by passing dry nitrogen through a urea-methanol solution. The details of each of these methods are described in the succeeding sections. A positive humidity-dependence was established for the urea measurements taken with the instrument detailed in this study (*Figure S7*). This relationship was then used to provide humidity-corrected quantitative data from the airborne measurements. All of the data used to provide the calibrated measurements were normalised to per one million of the sum of the reagent ions, I^- and $[I.H_2O]^-$.

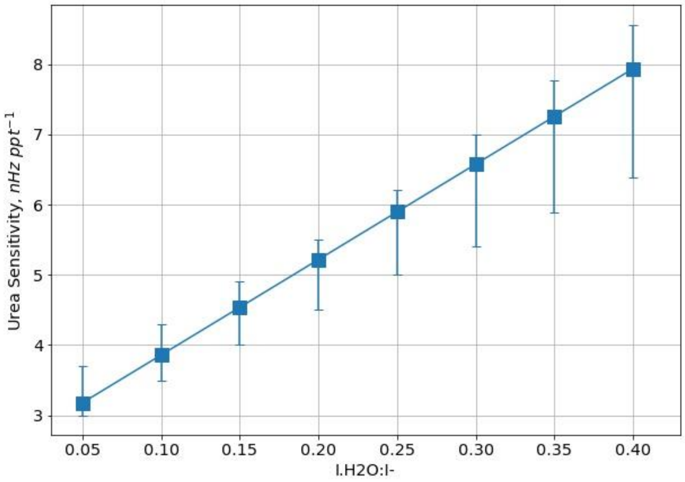

Figure S7: Sensitivity of I-CIMS used in this study to urea covering the range of I:I.H2O observed in flight conditions. The instrument shows a strong positive humidity dependency which was used to provide humidity corrected quantitative data. The sensitivity values and measurement data are normalised to per one million of the sum of the reagent ions, I^- and [I.H2O].

FIGAERO-CIMS

The University of Manchester FIGAERO-CIMS has been previously described in detail by (8) and is used here for calibration of the flight CIMS data. Briefly, The FIGAERO inlet provides molecular determination of gas- and particle-phase samples. In normal operation during the gas-phase measurement mode, particles from the aerosol sample are collected on a PTFE filter. After a period of collection, the filter is moved to the inlet of the instrument, and dry, heated nitrogen is passed through it to vaporize the particulate for analysis by the ToF-CIMS. The evolution of the MS signals from different compounds changes independently as a function of temperature, creating a thermogram that is m/z specific. The analysis software and procedure described above for in-flight sampling is then utilised to attain high-resolution 1 Hz time series.

In this study, for each sample, a known concentration of urea (Sigma Aldrich, 99% purity) dissolved in methanol (Sigma Aldrich, 99.8% purity) was placed on a new filter using a microliter syringe and in the FIGAERO. A temperature controlled nitrogen flow (2 SLM) was then delivered across the filter, this is known as the 'temperature ramp' phase. During this period the filter was ramped to 200 °C (temperature above the filter) over a period of 20 minutes (at a rate of 8.75 °C min^{-1}) and then held at this temperature for 15 minutes, known as the 'temperature soak' phase, and then finally cooled back down to 25 °C over a period of 15 minutes. The instrument humidity was controlled by the addition of water vapour, generated by flowing dry nitrogen through deionised water at flows ranging from 0-100 sccm, behind the filter and directly into the IMR region. Samples were taken at a range of humidities within the normal aircraft $I.H_2O:I^-$ working range (<0.10-0.40). Blank filters were routinely run as a background measurement.

For each sample, the urea signal is then integrated over the full heating period (i.e. temperature ramp and soak) and the nearest integrated blank urea signal was subtracted from integrated sample signal. It is assumed that almost all of the urea has been volatilised off the filter during this period (*Figure S8*). Sensitivity values were then yielded from the known urea concentration flowed for each sample and the corresponding signal intensity of urea. Samples of multiple concentrations were also run at a single humidity, which yielded a linear response and the same sensitivities. Additionally, the signal for isocyanic acid (HCNO) was monitored using the CIMS as it generally assumed that urea decomposes to HCNO upon heating. In our experiments, the HCNO signal represented less than 1% of the urea signal (*Figure S8*).

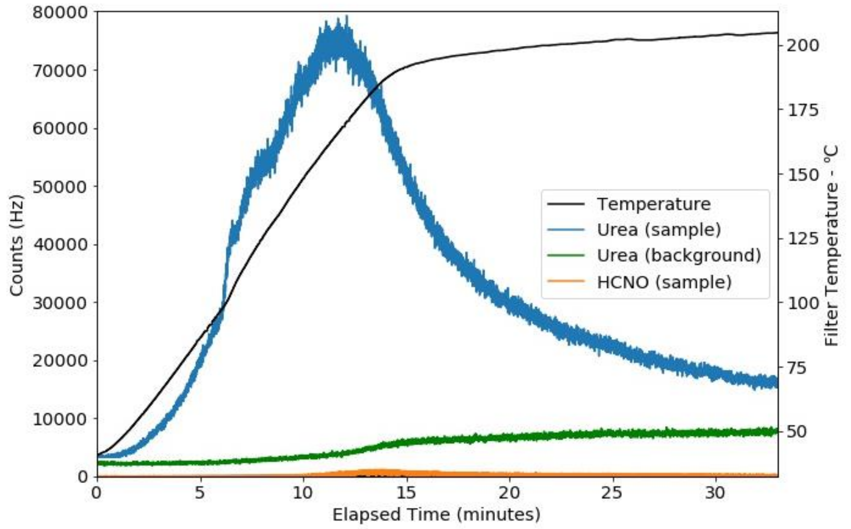

Figure S8: Example thermogram from a urea sample using the FIGAERO-CIMS. The signal for isocyanic acid (HCNO) was also monitored as a tracer for the thermal decomposition of urea. In our data the signal for HCNO represented less than 1% of the urea signal. The signal for urea during the nearest background filter is also shown.

Bubbler Experiments

A constant flow of urea was generated by flowing 100 sccm of dry nitrogen through a urea-methanol solution (urea; 99.9% purity, Sigma Aldrich, methanol; 99.8% purity, Sigma Aldrich) mixed into a nitrogen flow with varying humidities in programmable steps to control the $I.H_2O:I^-$ ratio within the maximum range observed during the reported flights (*Figure S9*). The response of the urea signal to changes in humidity was monitored and analysed to produce normalised 1 Hz time series as previously described. Solutions of pure methanol were also checked to ensure the solvent was not contaminated with urea. During these experiments all flows were drawn through 1/4″ PTFE lines and with the IMR pressure actively set to 72 mbar.

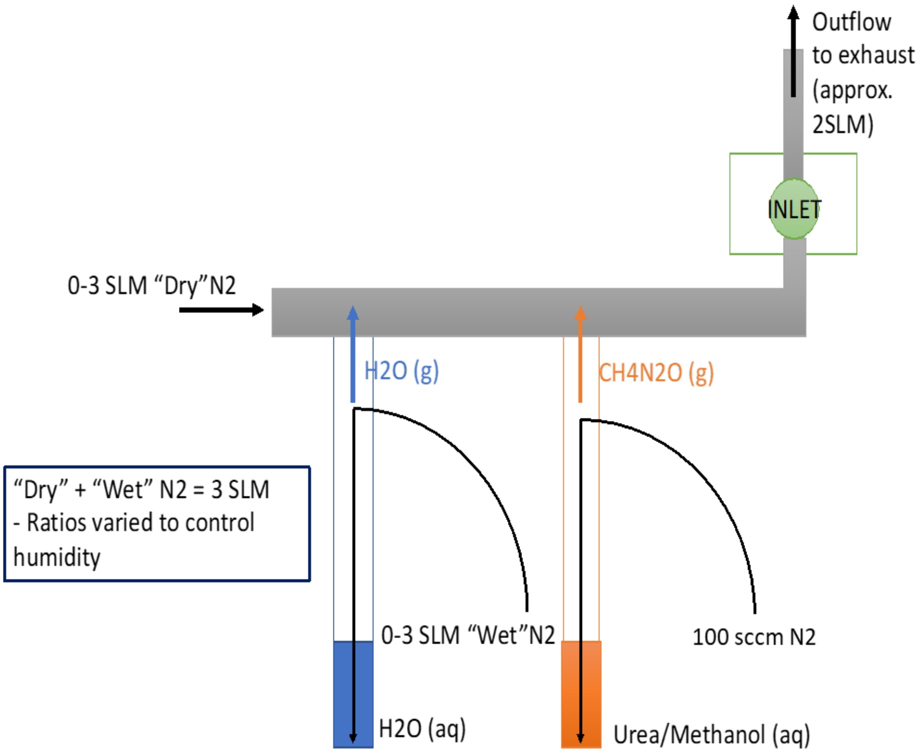

Figure S9: Experimental set-up used to produce a constant flow of gas-phase urea into the instrument during calibration experiments

Sensitivity Determination

The humidity-gradient determined from each of the respective methods, FIGAERO and bubbler, were compared. However, the yielded relationship from the bubbler experiments is not quantitative and so is used in this study to provide confidence in the results of the FIGAERO experiments. The bubbler results were found to fall within the 95% confidence intervals of the FIGAERO data, and to validate the novel FIGAERO derived humidity dependency. The average humidity-gradient from the two respective methods was used for the absolute humidity-dependence for the I-CIMS in this study. Whilst the exact IMR conditions on the aircraft cannot be absolutely replicated with the FIGAERO, gas-phase sensitivities with various calibration standards e.g. formic acid from known concentration gas mixtures between the two setups are within the reported experimental

error. The error from the determined sensitivity values is 5-20 %, with the largest uncertainty at the upper-end of the instrument humidity range. The combined largest error, from the peak identification and sensitivity values, for the reported urea mixing ratios is therefore estimated to be 8-23 %.

Additional aircraft measurements (CO, O_3, NOx, HCN and Black Carbon)

Measurements of CO dry-air mole fractions were sampled using an Aerolaser AL5002 Vacuum-UV fast fluorescence instrument. Specifics about the principles of operation for this instrument are provided by (9). The total 1σ at 1 Hz precision for airborne CO measurements is estimated to be ± 1.8 ppb at 100 ppb mixing ratio (typical background CO mixing ratio in the free troposphere), with an overall uncertainty of ± 2.7 ppb (or 2.4 %), whichever is greatest (10). However we recently discovered that a faulty inlet drier may have impacted the accuracy of our CO measurements in 2017–2019 and yielded a +9 ± 9 ppb bias in our data for ACSIS4-5 and MOYA-II. The CO data in this study was used to determine a statistical threshold for each campaign for excluding urea measurements with potential anthropogenic input during the ACSIS flights (see *Supplementary Information, section 3*). As a statistical approach was taken, the positive bias is not expected to affect this data filtering step. Furthermore, the calculated ER, EF and MCE values rely on a change in the CO measurement and as such any CO measurement systematic positive off-set would cancel out and not affect the calculated values.

O_3 concentrations were measured using a UV photometric analyser (model TEi-49i, Thermo Fisher Scientific Inc., USA), with a precision of 0.3 ppbv and overall uncertainty of 4%. Both the Aerolaser CO and the TEi ozone instruments were mounted within the pressurised cabin of the aircraft within a single 19" rack. Air was sampled by means of a window-mounted rearward facing inlet comprising of 1/4" PFA tubing housed within 3/8" stainless steel tubing.

In-situ measurements of NO were made using a custom built chemiluminescence instrument with NO2 measured by photolytic conversion at 385 nm to NO on a second channel following the design of Pollack (11). In-flight calibrations for NO sensitivity and NO_2 conversion efficiency were carried out a minimum of three times per flight by standard addition of 5 ppmV NO in nitrogen (BOC) to the sample inlet resulting in a calibration concentration of ~5.1 ppbV. NO_2 conversion efficiency was determined by gas phase titration of a portion (~90%) of the NO standard with Ozone generated from pure O_2 by low pressure mercury discharge lamp. The calibration factors were interpolated throughout the flight to account for any sensitivity drifts in the instrument. The chemiluminescent zero was determined every 5 minutes and also interpolated between. 3σ detection limits were ~30 pptv for NO and ~60 pptv for NO_2 for 1 Hz data, with root sum square uncertainties of ~17% for NO at 0.1 ppbv and ~23% for NO_2 at 0.1 ppbv.

Measurements of HCN were made using the iodide CIMS detailed in this study and as described in detail by (10).

Refractory black carbon (rBC) concentrations were measured using a single-particle soot photometer (SP2), the instrument setup, operation and data interpretation procedures of which on the ARA have been described by (12). The SP2 consists of four optical detectors and one Nd:YAG crystal laser with a Gaussian intensity distribution. It can detect BC-containing particles with an equivalent spherical diameter in the range of 70 – 850

nm (13). Briefly, the laser beam at λ = 1064 nm heats particles containing absorbing rBC material to their incandescence temperature, and visible light is emitted. Two detectors in the SP2 will capture the incandescence signal, which is proportional to the mass of rBC present in the particle, regardless of mixing state. Aquadag rBC particle standards were used to calibrate the SP2 incandescence signal during the campaign, following the calibration procedures in (14).

Section 2: Model Simulations

STOCHEM-CRI is a semi-Lagrangrian model where the tropospheric layer of the atmosphere is divided into 50,000 constant mass air parcels. The model allows computationally efficient simulation of chemical processes within the air parcel, alongside the emission and removal processes, to be uncoupled from the transportation of the parcel. Within the model both physical and chemical processes are driven by meteorological archived data from the U.K. Meteorological Office (UKMO) Unified Model. The UKMO model operates across a grid resolution of 1.25^o longitudes by 0.83^o latitude and 12 unevenly spaced vertical levels, with the upper boundary up to 100 hPa (15). The model and its meteorological parameterizations were briefly described in (16) and updated by (17). The model employs a comprehensive reduced chemical scheme, known as the Common Representative Intermediate version 2 Reduction 5 (CRI-v2-R5; (18–20)) with the updated isoprene degradation mechanism (CRI-v2.2; (21))which is traceable to the Master Chemical Mechanism (MCM) (www.mcm.leeds.ac.uk). The CRI allows complicated and identifiable hydrocarbons to be included in atmospheric models and for their degradation chemistry. The output data from the model run has a resolution of 5° longitude by 5° latitude and has 9 vertical levels, which span from the surface up to an altitude of ~15 km.

A suite of model simulations were conducted to assess (i) the possible source signature of urea, (ii) the impact of individual loss processes on the mixing ratios of urea, (iii) the global burden of urea and its contribution to the total reduced nitrogen. The measurement data (this study) shows the abundances of urea in the marine boundary layer, thus the possibility of the oceanic emissions of urea has been investigated by modelling urea emissions with a similar spatial and temporal distribution as that of dimethyl sulfide (DMS) from the ocean with the 2-fold (oceanic) emission total (56.2 Tg N/year; (22)). Oceanic emissions of DMS are distributed using a two-dimensional source map at a resolution of 5° longitude × 5° latitude taken from the EDGAR database (23). The loss of urea by reaction with OH (1.3×10^{-12} molecule^{-1} cm^3 s^{-1}) was accounted for in the model using estimated rate coefficients from the EPI SuiteTM software version 4.11 (www.epa.gov). The depositional parameters (deposition velocities over land and ocean, scavenging coefficients) of dry deposition and wet deposition of urea used in the model are assumed to be the same as NH_3. The wet depositional loss of urea is assumed to be more significant than the chemical loss, so we investigated it's impact by running the model without wet deposition loss process referred as OCEAN_WOWD.

Model results:

The model simulations are compared with the ACSIS campaigns (ACSIS-4, ACSIS-5, ACSIS-6, ACSIS-7) only due to the limited spatial range of the ACRUISE flights (*Figure S10*). Simulated concentrations of urea were found to be in reasonable agreement with measurements, with an average model bias of -33 pptv for all four campaigns (*Figure S10*). As the measurements for ACSIS-7 did not go above the limit of detection the bias was calculated using a range from 0-30 pptv. The model significantly overestimated the urea mixing ratios during the ACSIS-7 campaign with an average bias of +73 pptv. This suggests that at this time period DMS is not a suitable tracer for urea emissions and so are not included in the analysis discussed subsequently. Excluding this campaign, the simulated concentrations were found to be in reasonable agreement with measurements, with an average model bias of -71 pptv. For the measurements during February (ACSIS-4 and ACSIS-6) the model-measurement agreement is good with an average bias of -23 pptv. However, the model could not capture the high measured urea during August (ACSIS-5) with an average bias of -167 pptv suggesting a large missing emission flux in the model. Excluding the biomass burning events in the measurement data (ACSIS-6; *see Figure S10c*) improves the agreement between model-measurement with the bias reduced from +10 pptv to -2 pptv. There is a consistent bias throughout the troposphere with -42 pptv in the lower troposphere (0-5 km) and -23 pptv in the mid troposphere (6-8 km), with the difference between the observations and the model during ACSIS-5 contributing the most to this bias (-214 pptv in the lower troposphere and -50 pptv in the mid troposphere). In general, the simulated urea concentrations with and without wet deposition bracket the measurements except for the ACSIS-5 data (*Figure S10*). Since we anticipate that urea will be less soluble than NH_3, this result adds weight to the preliminary model results. Excluding the wet deposition in the simulation OCEAN_WOWD, the best agreement is found (bias: -2 pptv) in the lower troposphere, but a large overprediction of model is found (bias: +45 pptv) in the upper troposphere suggesting that wet deposition is the largest sink for urea and controls its concentration in the free troposphere. The model profiles exhibit near surface gradients much steeper than the measurements which could be due to the cloud effects on the wet depositional loss. There were few clouds during the measurement periods, however, the coarse meteorological grids of the model may have been sampling cloud all the time resulting in an overprediction of the wet deposition removal. Some episodes show that rapid uplift is causing the elevated levels that cannot be modelled at present.

The annual tropospheric global burden and lifetime of urea are found to be 0.16 Tg N and 1.0 day, respectively, for the OCEAN case scenario. The results from the OCEAN simulation demonstrated significant wet deposition loss (~60%). Removing wet deposition of urea in OCEAN _WOWD increases the global burden to 0.82 Tg N and lifetime to 5.4 days, showing the significance of the physical loss processes of urea.

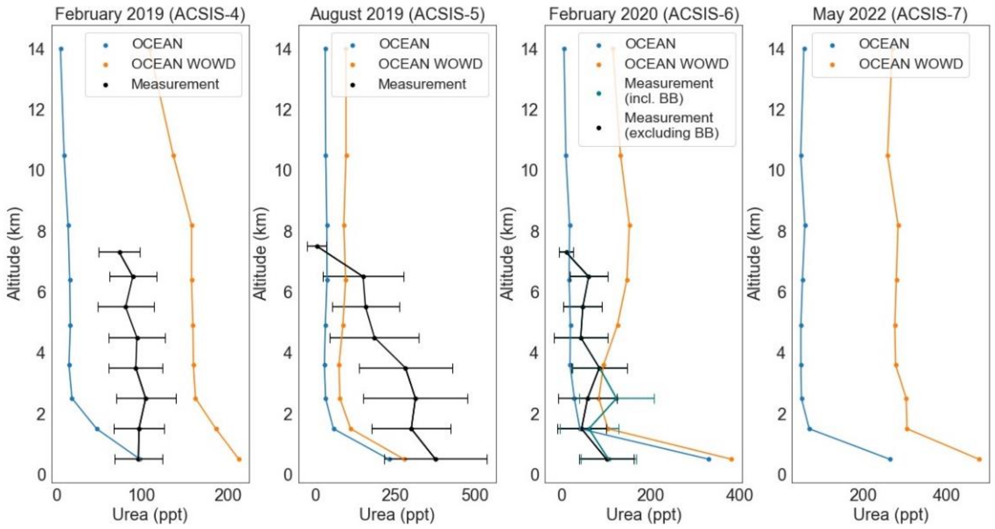

Figure S10: Comparison of measured urea from panel a) ACSIS-4 (February 2019), b) ACSIS-5 (August 2019),c) ACSIS-6 (February 2020), with and without biomass burning (BB) event and d) ACSIS-7 (May 2022), with model urea simulated by OCEAN and OCEAN_WOWD. The black lines represent measurement data and the error bars for measurement data represent one standard deviation of uncertainty. No measurements are shown for ACSIS-7 as these did not exceed the limit of detection.

Table S1: Global marin budget and calculated tropospheric lifetime of urea from different simulations and ammonia (NH_3)

	Urea OCEAN	Urea OCEAN_ WOWD	NH_3
**Emissions (Tg N/yr)**	56.2 ± 6.5	56.2 ± 6.5	8.2 ± 0.7
**Losses (Tg N/yr)**			
Reaction by OH	5.3 ± 0.8	21.4 ± 2.1	0.4 ± 0.0003
Dry deposition	17.5 ± 3.0	34.6 ± 6.0	1.9 ± 0.03
Wet deposition	33.2 ± 4.0	0	2.7 ± 0.05
Loss to NH_4^+	-	-	3.1 ± 0.06
**Global burden (Tg N)**	0.16 ± 0.02	0.82 ± 0.13	0.021 ± 0.003
**Lifetime (days)**	1.0 ± 0.1	5.4 ± 0.4	0.9 ± 0.1

Note: All values have been shown as average ± 1 SD for the twelve months data

Section 3: Supporting Analysis

Identification of clean marine air masses

To remove potential interference of anthropogenic contributions of urea to the marine environment and identify air masses representative of clean marine air masses, data was excluded if concentrations of CO and NO_x exceeded a set threshold. For each campaign, except ACRUISE-2, a median value for CO and was calculated and periods where CO exceeded this value and NOx levels exceeded 50 pptv were excluded from the ϑq analysis and the urea mixing ratios reported in '*Results: A significant ocean source of urea'*. For flights C202, C211, C216 and C224 only the CO threshold was applied as NOx data was unavailable. For the ACRUISE-2 flights a threshold using rBC concentrations was used as CO and NOx data was unavailable. Measurements were excluded when the rBC mass concentration exceeded 20 ng m^{-3}.

Calculation of boundary layer turnover time

To a first approximation, the time taken for one turnover in the boundary layer can be calculated using the following the equation:

$$Time\ (sec) = \frac{1}{10}\bar{\mu}.h$$

Where $\bar{\mu}$ is the horizontal wind speed (m s^{-1}) and h is height of the boundary layer (m).

Identification of biomass burning plumes (C218, C219, C221, C223 and C224)

A statistical threshold approach, as determined and described by (24) for the data used in this study, was used to determine when a biomass-burning plume was being sampled. A seven standard deviation enhancement above background in both CO and HCN was used as the threshold.

To calculate the change in the urea mixing ratios in the identified plumes a background value was estimated for each flight. A background value was taken for above and below the biomass-burning plumes and averaged to give a single value for each flight and then applied to the time series at each time point in the identified plumes (*Figure S11*). A threshold of a maximum of three standard deviation above background in CO and HCN was used to determine air masses that are assumed to be independent of the biomass-burning plumes. The above plume background region was defined from the altitude at which this threshold was met, above the plume, and up to 500m beyond this point. The average urea concentration within this region was taken as the above plume background value. For the below plume background value, an average value was taken between one and three standard deviations above background in CO and HCN for air masses below the plume. A one standard deviation threshold was used as an approximation to being above the marine BL. For comparison, the calculated background values were applied to 'clean free troposphere' air masses sampled within a flight's individual altitude range of the biomass-burning plumes. Air masses were classed as 'clean free troposphere' if they met the threshold for being independent of biomass-burning plumes. Flights C220 and C222 were excluded from the biomass burning analysis. Flight C220 was disregarded as the urea mixing ratios were continuously enhanced

rather than two discrete plumes as observed in other flights, one at the surface and one in the biomass burning plume. During flight C222 no biomass burning plumes were identified by the statistical threshold method.

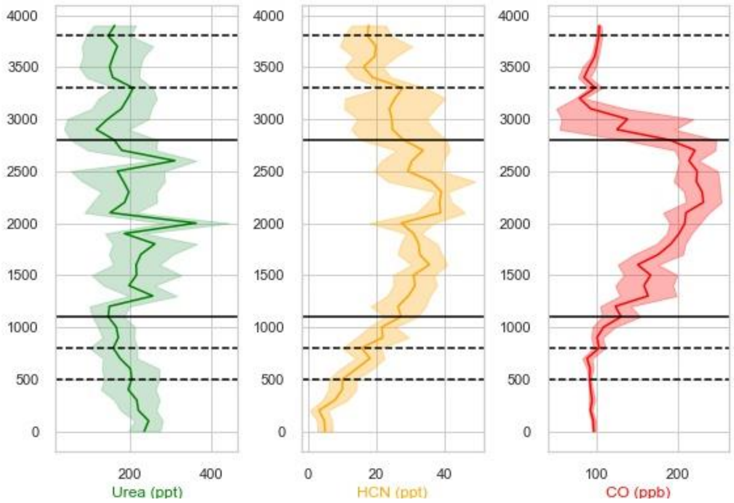

Figure S11: Illustration of background values determined for calculating urea enhancements within biomass-burning plumes. Measurements between the solid black lines are those identified as being in biomass-burning plumes by the statistical threshold. Measurements between the dashed lines represent those identified as background regions and an average value was yielded from the upper and lower regions.

Calculation of fire enhancements factors during MOYA-II

The fire plumes presented in this study and sampled over Uganda during the MOYA-II flights (C127-C134) have been described by (10) who determined emission ratios and enhancement factors (EFs) for a wide range of trace gases. The same methodology is applied here to determine EF values for urea in near-field fires. EFs are a measure of the mass of a species emitted (in grams) per kg of dry matter burnt. When compared with modified combustion efficiency (MCE) values, the trend between the two parameters can reveal important information as the proportion of trace gases emitted typically depends on the completeness of combustion.

Figure S12 shows a time series of urea, alongside HCN and CO, during a flight designed to sample fresh fire plumes that by visual inspection appeared to be mainly from burning tropical grasses (10). The fire plumes can be identified by the sharp enhancements in HCN and CO which also show enhancements in urea, reaching a maximum of over 1.7 ppb. A single value for smouldering (MCE < 0.90) and for flaming (MCE > 0.90) fires (25) was calculated from the fire plumes sampled during MOYA-II. Smouldering fires (EF: 0.059, range (r)=0.063-0.085 g kg^{-1}; MCE: 0.86, r=0.86; *n=2*) gave the highest EF compared with those classed as flaming (EF: 0.014, r=0.0042-0.031 g kg^{-1}; MCE: 0.94, r=0.90-0.96; *n=13*). These are the first EFs reported for urea but are consistent with the expectation of increased amounts of reduced products being emitted from fires with a higher degree of smouldering. Furthermore, urea pyrolysis typically occurs at temperatures above 150 °C (26) and would be less complete in a lower MCE fire. Typical EFs for other nitrogen species emitted from savannah and grassland burning, the assumed dominant fuel source during MOYA-II, range from 2.5 g kg^{-1} for NOx, 0.89 g kg^{-1} for NH_3 and 0.17 g kg^{-1} for N_2O (27).

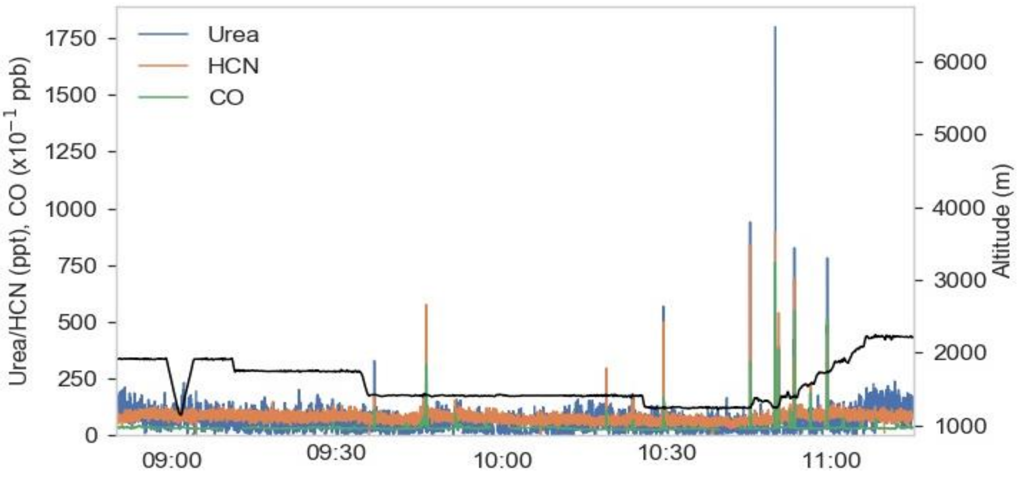

Figure S12: Time series showing enhancements of urea in fresh biomass burning plumes. The measurements for urea and the biomass burning markers HCN and CO are shown for a flight targeting fire plumes that by visual inspection appear to be burning of maize crops. The enhancements in CO and HCN indicate the location of these fire plumes and also indicate biomass burning to be a source of urea.

Air mass back trajectory analysis

Air mass back trajectories were simulated from the position of the aircraft every 30 seconds using the HYSPLIT (Hybrid Single-Particle Lagrangian Integrated Trajectory; http://www.arl.noaa.gov/ready/hysplit4.html) model. Each simulation was run for 120 hours with GDAS 1° meteorological data to give back trajectories with meteorological variables (potential temperature and rainfall).

Confined marine boundary layer thermodynamic profiles

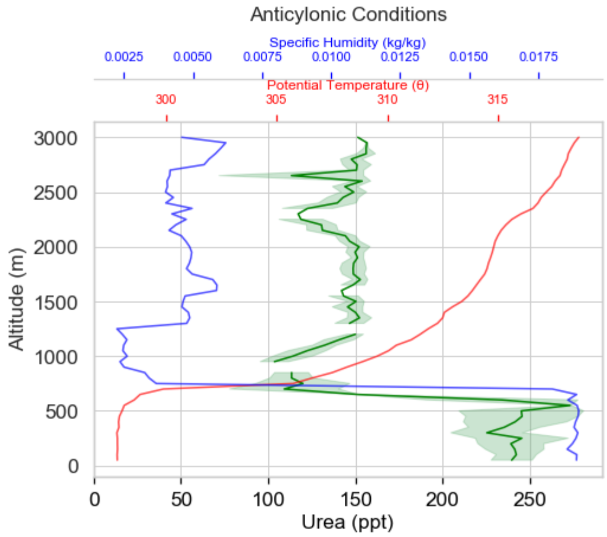

Figure S13a (left): Thermodynamic profile alongside urea concentrations during a profile to the sea-water surface in anticyclonic conditions

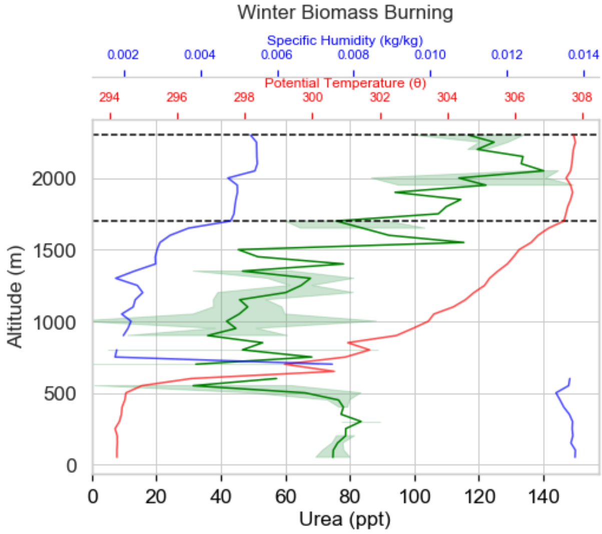

Figure S13b (right): Thermodynamic profile alongside urea concentrations during a profile to the sea-water surface in the tropical Atlantic during a period when biomass burning dominates the region. The biomass burning layer is indicated by the dashed black lines.

Source contributions during ACSIS-6

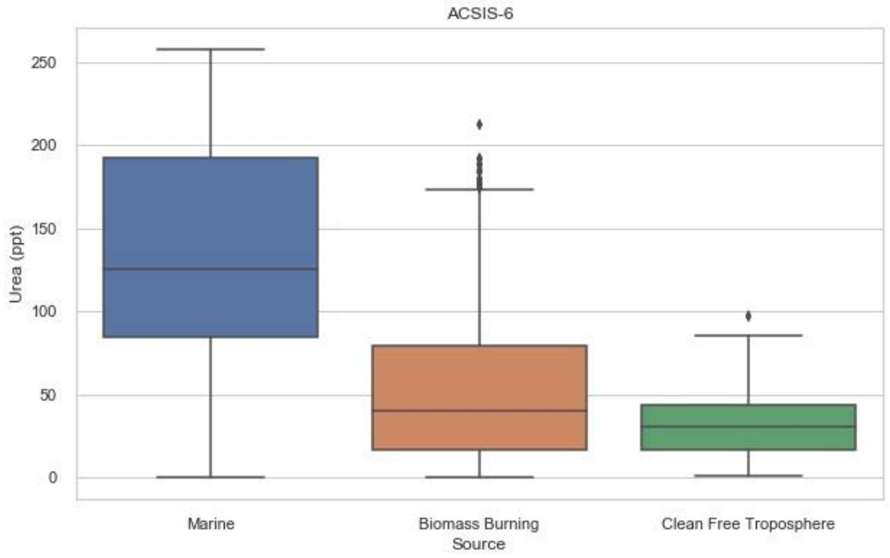

Figure S14: Measurements made during ACSIS-6 identified a secondary source of urea to the remote marine environment – biomass-burning plumes. The biomass-burning associated concentrations are shown alongside clean air masses of a similar altitude ('Clean Free Troposphere') for comparison and show that urea concentrations are elevated aloft in the presence of biomass-burning plumes. The highest concentrations in these instances are observed at the surface and originate from the sea-surface ('Marine').

Comparison and calculation of urea equilibrium partitioning concentrations

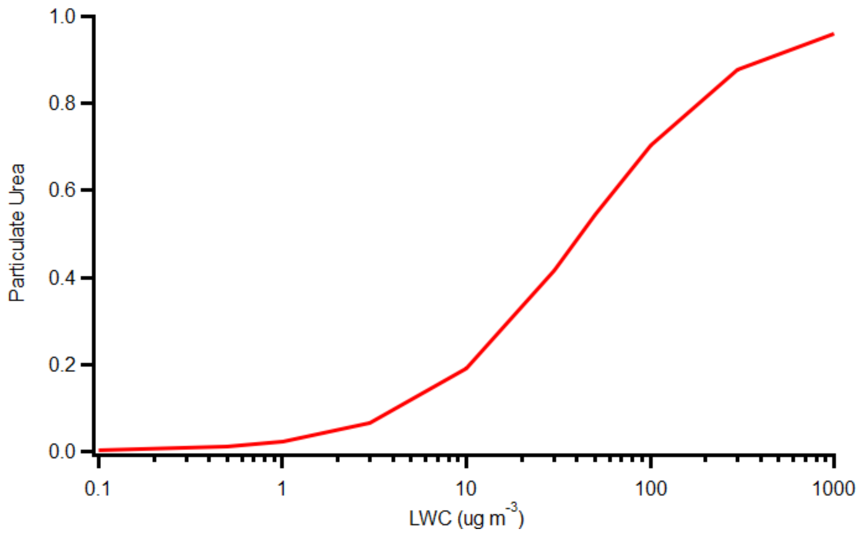

Figure S15: Fraction of urea in the particulate phase as a function of liquid water content (LWC) at equilibrium with the gas phase for a Henry's law constant of 1×10^{9} M atm^{-1} (28)

$$concentration\ of\ urea\ in\ liquid\ water = \frac{concentration\ of\ urea\ in\ particulate\ phase}{LWC\ in\ air} \quad \text{(Eq. 1)}$$

$$where$$

$$Liquid\ water\ content\ (LWC)\ in\ air = \frac{LWC\ in\ aerosol\ (aLWC)}{\rho(water)} \quad \text{(Eq. 2)}$$

$$\rho(water) = 1000\ g\ L^{-1}$$

Table S2: Calculation of urea concentration in the liquid phase from the gas phase observations

		Reference
Median urea concentration for all flights (below 1 km)	90 pptv<br>0.24 ug m^{-3}	This study
Typical liquid water content for a marine aerosol (aLWC)	10 ug m^{-3}	(29)
Fraction of urea in particulate phase	0.2	From Figure S15
Calculated concentration of urea in particulate phase	0.06 ug m^{-3}<br>0.001 umol m^{-3}	

LWC in air	1 x 10^{-8} L m^{-3}	From Eq. 2
**Calculated concentration of urea in aerosol liquid water**	**0.1 mol L^{-1}**	**From Eq. 1**

Table S3: Calculation of urea concentration in the liquid phase from the literature particulate concentrations

		Reference
Typical atmospheric concentrations of particulate urea	0.2-17.7 nmol N m^{-3}	(30)
Average urea particulate concentration	9 nmol N m^{-3}<br>39 nmol m^{-3}	(30)
aLWC for marine aerosol	10 ug m^{-3}	(29)
LWC in air	1 x 10^{-8} L m^{-3}	From Eq. 2
**Calculated concentration of urea in aerosol liquid water**	**3.9 mol L^{-1}**	**From Eq. 1**

Considering the highly variable concentrations, over an order of magnitude, for both urea in the particulate and gas phase, we show that that the gas phase measurements presented in this study are consistent with equilibrium partitioning based on previous measurements of particulate urea in the marine environment (30) and typical aLWC of marine aerosols. However, as shown in the calculations below, the literature particulate concentrations are inconsistent with literature seawater surface concentrations.

Comparison with literature seawater surface concentrations (bubble bursting)

$$\text{Concentration of NaCl in sea water } \approx 35 \text{ parts per } 1000$$

$$\text{Concentration of water in sea water } \approx 965 \text{ parts per } 1000$$

$$\text{Equilibrium growth factor of a dry NaCl particle by mass } \approx 10$$

$$\text{Ratio of dry salt to wet mass is } 1:10$$

Therefore, if a drop were ejected from the seawater surface into the atmosphere the concentration of solutes would increase by approximately a factor of 100-1000

$$Seawater\ urea\ concentration\ for\ open\ ocean \ \ \approx 300\ nmol\ L^{-1} \quad (31)$$

$$Urea\ concentrations\ in\ sea\ salt\ particles\ (i.e. aerosol) \approx 30 - 300\ umol\ L^{-1}$$

This suggests that concentrations of urea in the surface seawater would need to be approximately 3-30 mmol L^{-1} to explain the atmospheric observations from a bubble bursting mechanism.

Calculation of required seawater concentrations for direct air-sea gas exchange

The concentration of urea in the surface seawater required to sustain a flux of 56.2 Tg N yr^{-1} (122.2 Tg urea yr^{-1}) from air-sea gas exchange is calculated using the 'two-film' model of air-sea gas exchange according to (32) and assuming an oceanic surface area of 3.6 x 10^{18} cm^2 (32).

$$F = -k_a\{[Urea_{(g)}] - K_H[Urea_{(sw)}]\} \qquad (Eq.\ 3)$$

where *F* is the ocean atmosphere flux (nmol m^{-2} s^{-1}), k_a is the gas phase transfer velocity (m s^{-1}), K_H is the dimensionless Henry's law coefficient for urea ($4.03\text{x}10^{-11}$; (28)), [$urea_{(g)}$] and [$urea_{(sw)}$] are the concentrations of urea in the gas-phase and seawater respectively and must be in the same units (in this study nmol m^{-3}). The gas transfer velocity (k_a) was parametrised as derived by (33) and calculated as a function of the wind speed 10m above the water surface (U_{10}).

$$k_a = 1 \times 10^{-3} + \frac{u_*}{13.3S_{c_a}^{\frac{1}{2}} + C_D^{-\frac{1}{2}} - 5 + \frac{\ln(S_{c_a})}{2\kappa}} \qquad (Eq.\ 4)$$

Where κ is the von Karman constant, taken to be 0.4 in seawater (33), S_{c_a} is the Schmidt number in air, C_D is the drag coefficient and is related to the friction velocity, u_*:

$$C_D = (\frac{u_*}{u_{10}})^2 \qquad (Eq.5)$$

$$u_* = u_{10}\sqrt{6.1 \times 10^{-4} + 6.3 \times 10^{-5}\, u_{10}} \qquad (Eq.\ 6)$$

The Schmidt number in air is the ratio of the kinematic viscosity of air (v_a) and the diffusivity of the gas of interest in air (D_a). v_a is the ratio of the dynamic viscosity of air (n_a) and the density of air (ρ_a). n_a and ρ_a are calculated according to (34).

$$S_{c_a} = \frac{v_a}{D_a} = \frac{n_a}{\rho_a D_a} \qquad (Eq.\ 7)$$

The D_a of urea is calculated according to (35):

$$D_a = 0.001 \times T^{1.75} \frac{M_r^{\,0.5}}{[\left(PV_A^{\frac{1}{3}}\right) + V_B^{\frac{1}{3}}]^2} \qquad (Eq.\ 8)$$

Where, T is the temperature in °C, P is the pressure in atm (assumed to be unity in this study), V_a is the molar volume of air (assumed here to be 20.1 cm^3 mol^{-1}), V_b is the molar volume of the gas of interest (i.e urea), M_r is a function of the relative molecular masses of air (M_a), assumed to be 28.97 (36) and of the gas of interest, urea (M_b):

$$M_r = \frac{M_a + M_b}{M_a M_b} \quad \textit{(Eq. 9)}$$

Using the 'two-film' model, the required seawater concentrations to sustain a flux of 56.2 Tg N yr^{-1} (122.2 Tg urea yr^{-1}), taking a median gas concentration from the observations of 90 pptv (below 1km), and an average global wind speed over the ocean (7.4 m s^{-1}) is 171 mmol L^{-1}.

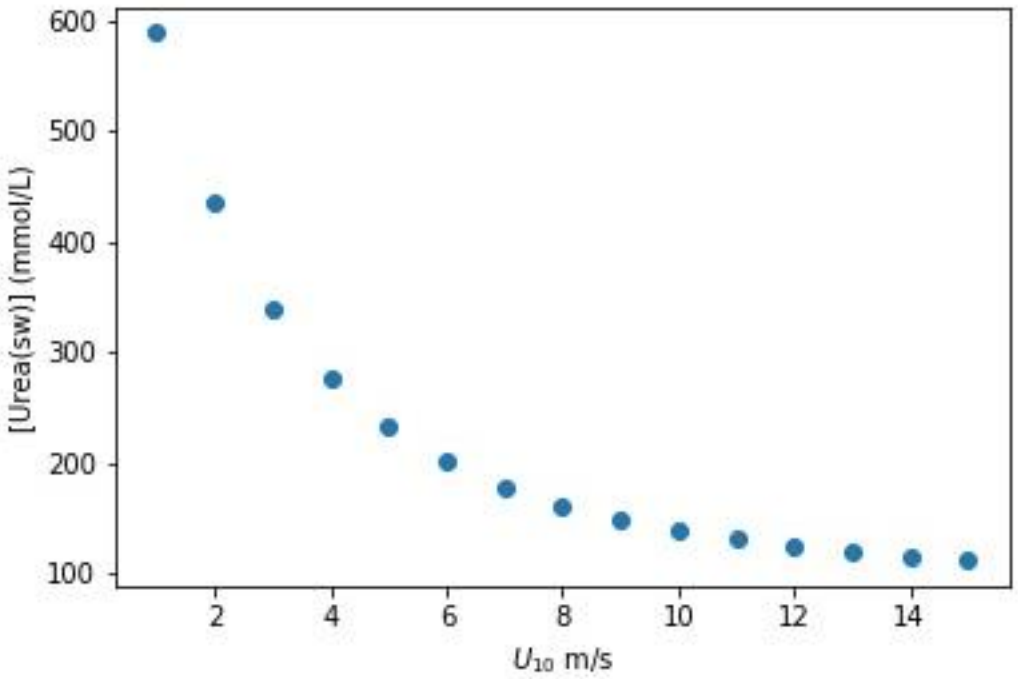

Figure S16: Required seawater concentrations to sustain a flux of 56.2 Tg N yr^{-1} as a function of wind speed

References

1. M. Priestley, *et al.*, Observations of Isocyanate, Amide, Nitrate, and Nitro Compounds From an Anthropogenic Biomass Burning Event Using a ToF-CIMS. *J. Geophys. Res. Atmos.* **123**, 7687–7704 (2018).

2. M. Priestley, *et al.*, Observations of organic and inorganic chlorinated compounds and their contribution to chlorine radical concentrations in an urban environment in northern Europe during the wintertime. *Atmos. Chem. Phys.* (2018).

3. M. Le Breton, *et al.*, Airborne observations of formic acid using a chemical ionization mass spectrometer. *Atmos. Meas. Tech.* **5**, 3029–3039 (2012).

4. B. H. Lee, *et al.*, Flight Deployment of a High-Resolution Time-of-Flight Chemical Ionization Mass Spectrometer: Observations of Reactive Halogen and Nitrogen Oxide Species. *J. Geophys. Res. Atmos.* (2018)

5. H. Stark, *et al.*, Methods to extract molecular and bulk chemical information from series of complex mass spectra with limited mass resolution. *Int. J. Mass Spectrom.* (2015)

6. M. J. Cubison, J. L. Jimenez, Statistical precision of the intensities retrieved from constrained fitting of overlapping peaks in high-resolution mass spectra. *Atmos. Meas. Tech.* (2015)

7. F. D. Lopez-Hilfiker, *et al.*, A novel method for online analysis of gas and particle composition: description and evaluation of a Filter Inlet for Gases and AEROsols (FIGAERO). *Atmos. Meas. Tech.* **7**, 983–1001 (2014).

8. T. J. Bannan, *et al.*, A method for extracting calibrated volatility information from the FIGAERO-HR-ToF-CIMS and its experimental application. *Atmos. Meas. Tech.* **12**, 1429–1439 (2019).

9. C. Gerbig, *et al.*, An improved fast-response vacuum-UV resonance fluorescence CO instrument. *J. Geophys. Res. Atmos.* **104**, 1699–1704 (1999).

10. P. A. Barker, *et al.*, Airborne measurements of fire emission factors for African biomass burning sampled during the MOYA campaign. *Atmos. Chem. Phys.* (2020)

11. I. B. Pollack, B. M. Lerner, T. B. Ryerson, Evaluation of ultraviolet light-emitting diodes for detection of atmospheric NO2 by photolysis - Chemiluminescence. *J. Atmos. Chem.* **65**, 111–125 (2010).

12. G. R. McMeeking, *et al.*, Black carbon measurements in the boundary layer over western and northern Europe. *Atmos. Chem. Phys.* (2010)

13. D. Liu, *et al.*, Size distribution, mixing state and source apportionment of black carbon aerosol in London during winter time. *Atmos. Chem. Phys.* (2014)

14. M. Laborde, *et al.*, Single Particle Soot Photometer intercomparison at the AIDA chamber. *Atmos. Meas. Tech.* (2012)

15. T. C. Johns, *et al.*, The second Hadley Centre coupled ocean-atmosphere GCM: Model description, spinup and validation. *Clim. Dyn.* (1997)

16. W. J. Collins, D. S. Stevenson, C. E. Johnson, R. G. Derwent, Tropospheric ozone in a global-scale three-dimensional Lagrangian model and its response to NO(x) emission controls. *J. Atmos. Chem.* (1997)

17. R. G. Derwent, *et al.*, Radiative forcing from surface NOx emissions: Spatial and seasonal variations. *Clim. Change* (2008)

18. M. E. Jenkin, L. A. Watson, S. R. Utembe, D. E. Shallcross, A Common Representative Intermediates (CRI) mechanism for VOC degradation. Part 1: Gas phase mechanism development. *Atmos. Environ.* (2008).

19. S. R. Utembe, L. A. Watson, D. E. Shallcross, M. E. Jenkin, A Common Representative Intermediates (CRI) mechanism for VOC degradation. Part 3: Development of a secondary organic aerosol module. *Atmos. Environ.* (2009)

20. L. A. Watson, D. E. Shallcross, S. R. Utembe, M. E. Jenkin, A Common Representative Intermediates (CRI) mechanism for VOC degradation. Part 2: Gas phase mechanism reduction. *Atmos. Environ.* (2008)

21. M. E. Jenkin, *et al.*, The CRI v2.2 reduced degradation scheme for isoprene. *Atmos. Environ.* (2019)

22. M. A. H. Khan, *et al.*, A modelling study of the atmospheric chemistry of DMS using the global model, STOCHEM-CRI. *Atmos. Environ.* (2016)

23. Olivier, J G.J., *et al.*, "Description of EDGAR version 2.0. A set of global emission inventories of greenhouse gases and ozone-depleting substances for all anthropogenic and most natural sources on a per country basis and on $1^o x 1^o$ grid" (1996).

24. J. D. Lee, *et al.*, Ozone production and precursor emission from wildfires in Africa. *Environ. Sci. Atmos.* **1**, 524–542 (2021).

25. J. S. Reid, R. Koppmann, T. F. Eck, D. P. Eleuterio, A review of biomass burning emissions part II: Intensive physical properties of biomass burning particles. *Atmos. Chem. Phys.* (2005)

26. P. M. Schaber, *et al.*, Thermal decomposition (pyrolysis) of urea in an open reaction vessel. *Thermochim. Acta* (2004)

27. M. O. Andreae, Emission of trace gases and aerosols from biomass burning - An updated assessment. *Atmos. Chem. Phys.* (2019)

28. R. Sander, Compilation of Henry's law constants (version 4.0) for water as solvent. *Atmos. Chem. Phys.* (2015)

29. K. Deetz, *et al.*, Aerosol liquid water content in the moist southern West African monsoon layer and its radiative impact. *Atmos. Chem. Phys.* (2018)

30. L. M. Zamora, J. M. Prospero, D. A. Hansell, Organic nitrogen in aerosols and precipitation at Barbados and Miami: Implications regarding sources, transport and deposition to the western subtropical North Atlantic. *J. Geophys. Res. Atmos.* (2011)

31. S. C. Painter, R. Sanders, H. N. Waldron, M. I. Lucas, S. Torres-Valdes, Urea distribution and uptake in the Atlantic Ocean between 50° N and 50° S. *Mar. Ecol. Prog. Ser.* (2008)

32. P. S. Liss, P. G. Slater, Flux of gases across the Air-Sea interface. *Nature* (1974)

33. M. T. Johnson, A numerical scheme to calculate temperature and salinity dependent air-water transfer velocities for any gas. *Ocean Sci.* (2010)

34. P. T. Tsilingiris, Thermophysical and transport properties of humid air at temperature range between 0 and 100 °C. *Energy Convers. Manag.* (2008)

35. E. N. Fuller, P. D. Schettler, J. C. Giddings, A new method for prediction of binary gas-phase diffusion coefficients. *Ind. Eng. Chem.* (1966)

36. W. A. Tucker, A. H. Nelken, "Diffusion coefficients in air and water" in *Handbook of Chemical Property Estimation Methods*, (1990).
